# Patterns of Admixture and Population Structure in Native Populations of Northwest North America

**DOI:** 10.1371/journal.pgen.1004530

**Published:** 2014-08-14

**Authors:** Paul Verdu, Trevor J. Pemberton, Romain Laurent, Brian M. Kemp, Angelica Gonzalez-Oliver, Clara Gorodezky, Cris E. Hughes, Milena R. Shattuck, Barbara Petzelt, Joycelynn Mitchell, Harold Harry, Theresa William, Rosita Worl, Jerome S. Cybulski, Noah A. Rosenberg, Ripan S. Malhi

**Affiliations:** 1 CNRS-MNHN-University Paris Diderot-Sorbonne Paris Cité, UMR7206 Eco-Anthropology and Ethno-Biology, Paris, France; 2 Department of Biochemistry and Medical Genetics, University of Manitoba, Winnipeg, Manitoba, Canada; 3 Department of Anthropology and School of Biological Sciences, Washington State University, Pullman, Washington, United States of America; 4 Departmento de Biología Celular, Facultad de Ciencias, Universidad Nacional Autonóma de México, Mexico City, Mexico; 5 Department of Immunology and Immunogenetics, Instituto de Diagnóstico y Referencia Epidemiológicos, Secretary of Health, Mexico City, Mexico; 6 Department of Anthropology, University of Illinois at Urbana-Champaign, Urbana, Illinois, United States of America; 7 Department of Anthropology, New York University, New York, New York, United States of America; 8 Metlakatla Treaty Office, Metlakatla, British Columbia, Canada; 9 Stswecem'c/Xgat'tem Band, British Columbia, Canada; 10 Splatsin Band Office, Enderby, British Columbia, Canada; 11 Seaalaska Heritage Institute, Juneau, Alaska, United States of America; 12 Canadian Museum of History, Gatineau, Quebec, Canada; 13 Department of Biology, Stanford University, Stanford, California, United States of America; 14 Institute for Genomic Biology, University of Illinois at Urbana-Champaign, Urbana, Illinois, United States of America; University of New Mexico, United States of America

## Abstract

The initial contact of European populations with indigenous populations of the Americas produced diverse admixture processes across North, Central, and South America. Recent studies have examined the genetic structure of indigenous populations of Latin America and the Caribbean and their admixed descendants, reporting on the genomic impact of the history of admixture with colonizing populations of European and African ancestry. However, relatively little genomic research has been conducted on admixture in indigenous North American populations. In this study, we analyze genomic data at 475,109 single-nucleotide polymorphisms sampled in indigenous peoples of the Pacific Northwest in British Columbia and Southeast Alaska, populations with a well-documented history of contact with European and Asian traders, fishermen, and contract laborers. We find that the indigenous populations of the Pacific Northwest have higher gene diversity than Latin American indigenous populations. Among the Pacific Northwest populations, interior groups provide more evidence for East Asian admixture, whereas coastal groups have higher levels of European admixture. In contrast with many Latin American indigenous populations, the variance of admixture is high in each of the Pacific Northwest indigenous populations, as expected for recent and ongoing admixture processes. The results reveal some similarities but notable differences between admixture patterns in the Pacific Northwest and those in Latin America, contributing to a more detailed understanding of the genomic consequences of European colonization events throughout the Americas.

## Author Summary

We collaborated with six indigenous communities in British Columbia and Southeast Alaska to generate and analyze genome-wide data for over 100 individuals. We then combined this dataset with existing data from populations worldwide, performing an investigation of the genetic structure of indigenous populations of the Pacific Northwest both locally and in relation to continental and worldwide geographic scales. On a regional scale, we identified differences between coastal and interior populations that are likely due to differences both in pre- and post-European contact histories. On a continental scale, we identified differences in genetic structure between populations in the Pacific Northwest and Central and South America, reflecting both differences prior to European contact as well as different post-contact histories of admixture. This study is among the first to analyze genome-wide diversity among indigenous North American populations, and it provides a comparative framework for understanding the effects of European colonization on indigenous communities throughout the Americas.

## Introduction

The population history of indigenous peoples of the Americas is of perennial interest to scholars studying human migrations. The Americas were the last continents historically peopled by modern humans, with recent evidence supporting an initial human entry via Beringia after the last glacial maximum [1]–[4]. Despite the absence of a deep written record, abundant archaeological sites and rich anthropometric, cultural, and linguistic variation in the Americas have long facilitated thriving programs of investigation of Native American population history and relationships [1], [5]–[8].

Population-genetic approaches applied to dense genome-wide datasets have recently expanded the forms of evidence available for studies of human migration [9]–[14]. In the Americas, genomic studies have been of particular value in understanding the diversity of admixture processes that indigenous communities have experienced with non-native populations following European contact [13], [15]–[21]. Studies have identified considerable variation in the level of admixture among populations, in the level of admixture among individuals within a population, in the contributions from different source populations, and in the magnitudes of the various ancestry contributions at different points in the genome [13], [15], [20]–[23].

Most of this genomic work has focused on populations in Latin America and the Caribbean, evaluating the demographic impact of colonizing individuals of European and African descent on local indigenous groups, and relatively few genome-wide investigations have been performed specifically on indigenous North American populations. Owing, in part, to differences in colonization practices between the British and French in North America and the Spanish and Portuguese in Central and South America, North America experienced a substantially different history of admixture [24]. In the Pacific Northwest, extensive contact with non-native populations began relatively recently, with the Russian expansion and the maritime fur trade in the 1700s. These events allowed indigenous communities to initially receive economic benefits from trade without the disruptive effects of colonization [25]. Multiple immigrant groups then entered the region in the 1800s, as Russian Alaska was transferred to the United States, and as borders between the United States and British-controlled Canada were settled. For example, Scandinavians migrating to Alaska were early contributors to the forestry and fishing industries [26]. In addition, the construction of the Canadian Pacific railway between 1881 and 1885 in British Columbia employed numerous Chinese and Japanese immigrants [27]. These immigrant groups had ample opportunity to intermarry with indigenous members of a variety of local communities.

To obtain a detailed picture of the genetic landscape of the Pacific Northwest of North America, we generated data on over 600,000 genome-wide single-nucleotide polymorphisms (SNPs) in 104 indigenous individuals from four coastal communities in Southeastern Alaska and British Columbia and two communities living in interior British Columbia. We combined these data with existing data from other geographic regions that together encompass 64 worldwide populations. This worldwide dataset allowed us to investigate the genetic structure of indigenous populations of the Pacific Northwest both locally and in relation to continental and worldwide geographic scales, and to further analyze the admixture landscape in the region. The results uncover both differences in the admixture patterns seen among indigenous Pacific Northwest populations as well as notable differences from comparable patterns observed in admixture studies of Latin America, illuminating differences in the histories of admixture experienced by Native American populations from across the American landmass.

## Results

**Figure 1:**
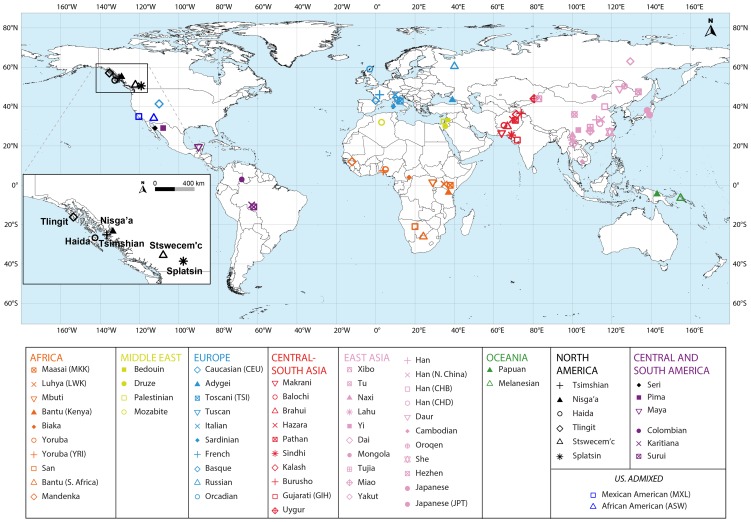
Map of populations included in the combined dataset. The Tlingit, Tsimshian, Nisga'a, Splatsin, Stswecem'c, and Haida populations, as well as the Northern Mexico Seri population indicated by a black diamond, were newly genotyped for this study. See Tables 1 and S1 for additional population information.

We genotyped 104 individuals from six native Pacific Northwest populations and one native population of Mexico at 616,794 SNPs. To assess the new samples in relation to relevant populations from other geographic regions, we integrated our new data with previously published standard data sets on 64 worldwide populations (Figure 1; Tables 1 and S1). After quality control, exclusion of related individuals, and reduction to a set of SNPs overlapping with the earlier datasets, our final dataset included 82 Pacific Northwest individuals, three Mexican Seri individuals and 2,055 individuals from 64 additional populations from Africa, Eurasia, Oceania, and the Americas (including two admixed populations from the United States) at 475,109 SNPs. We performed analyses of heterozygosity, population structure, and admixture, in each analysis focusing on the placement of the populations of the Pacific Northwest in relation to the other populations, both at a worldwide and at a continental scale.

**Table 1:** North American indigenous dataset.

Continental location	Population	Number of newly genotyped individuals	Number of unrelated individuals in the combined dataset	Latitude (°North)	Longitude (°East)	Distance to East Africa in kilo-meters^a^	Haplotype hetero-zygosity (SD)^b^	Missing data frequency (SD)^c^
NORTH-WESTERN AMERICA	Tsimshian	32	26	54.442	−130.433	14773	0.658 (0.012)	0.0003 (0.0007)
	Nisga'a	8	8	55.017	−129.547	14833	0.609 (0.017)	0.0118 (0.0235)
	Haida	12	10	54.017	−132.15	14857	0.687 (0.008)	0.0049 (0.0076)
	Tlingit	18	16	57.052	−135.339	15194	0.665 (0.010)	0.0068 (0.0132)
	Stswecem'c	15	13	51.593	−122.089	15311	0.654 (0.012)	0.0106 (0.0264)
	Splatsin	16	9	50.551	−119.140	15548	0.624 (0.012)	0.0131 (0.0297))
CENTRALAMERICA	Seri	3	3	29.017	−112.167	17846	0.572 (0.029)	0.0537 (0.0238)

a The distance to Addis Ababa along waypoint routes.

b Genome-wide mean haplotype heterozygosity and standard deviation across 22 chromosomes.

c The fraction of missing genotype data among the 475,109 total SNPs in the combined dataset, with the standard deviation taken across individuals within the population.

### Indigenous Northwest populations in the worldwide landscape of genome-wide diversity

#### Haplotype heterozygosity

**Figure 2:**
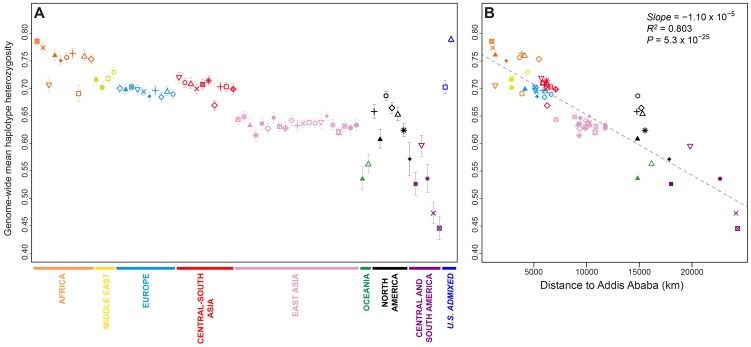
Genome-wide haplotype heterozygosities. (A) Mean expected haplotype heterozygosity in each population, with standard deviations across the 22 autosomes. (B) The correlation between mean haplotype heterozygosity and geographic distance from Addis Ababa. Population colors and symbols follow Figure 1.

Genome-wide mean haplotype heterozygosities in the indigenous populations from British Columbia and Alaska (mean = 0.649 with standard deviation [SD] = 0.028, across six populations; Table 1) did not differ significantly from those of East Asian populations (mean = 0.635 with SD = 0.009, across 21 populations; Wilcoxon two-sided test *P* = 0.194; Figure 2A and Table S1). They were, however, significantly higher than the heterozygosities of Central (mean = 0.565 with SD = 0.035, across the Seri, Pima, and Maya populations; *P* = 0.012) and South (mean = 0.485 with SD = 0.046 across the Colombian, Karitiana, and Surui populations; *P* = 0.012) American groups.

Population-level mean haplotype heterozygosities in the dataset ranged from 0.446 (SD = 0.021 across 22 chromosomes) in the Surui to 0.790 (SD = 0.006) in the African Americans. African populations had the highest heterozygosities (mean = 0.750 with SD = 0.030, across 10 populations), and South American populations had the lowest (mean = 0.485 with SD = 0.046). The admixed African American (ASW) and Mexican American (MXL) populations had relatively high heterozygosities, a likely result of admixture between differentiated ancestral sources.

Previous studies have reported a negative correlation between expected heterozygosity and geographic distance from East Africa [20], [28]–[31], consistent with an “Out of Africa” serial-founder model for human migrations [31], [32]. Addition of the Indigenous Northwest populations does not alter the general pattern of decline in haplotype heterozygosity with increasing distance from Addis Ababa (Figure 2B; slope = −1.10×10^−5^, *R^2^* = 0.803, *P* = 5.3×10^−25^). If we exclude the six Indigenous Northwest populations, however, the correlation increases (slope = −1.22×10^−5^, *R^2^* = 0.884, *P* = 2.8×10^−29^), suggesting that while heterozygosity in Indigenous Northwest groups is not incompatible with expectations from the serial-founder model, regional demographic mechanisms, such as peculiarities in migration routes, population-size fluctuations, and recent admixture, have likely had sizeable influences on genetic diversity in the Pacific Northwest. Note that although alternative haplotype block definitions change the scale of heterozygosity values, they have little effect on population patterns (Figures S1 and S2).

#### Population structure

**Figure 3:**
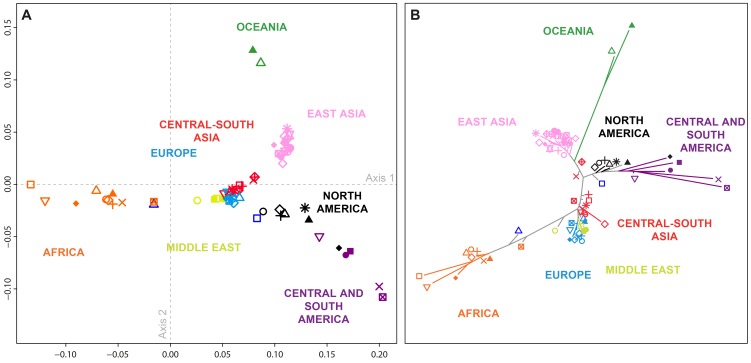
Population-level population structure for 71 populations. Shown are a (A) multidimensional scaling (MDS) plot (Spearman *ρ* = 0.965; *P*<10^−15^, comparing population-pairwise Euclidean distances on the two-dimensional MDS plot to their population pairwise *F*_ST_ values), and (B) Neighbor-joining tree based on population pairwise *F*_ST_. All the edges of the tree were supported by 100% of the 1,000 bootstrap replicates performed except for four edges corresponding respectively to the Kalash, Caucasian (CEU), Orcadian, and French populations (supported by 76%, 75%, 75%, and 74%, respectively, of the 1,000 bootstrap replicates). Population colors and symbols follow Figure 1.

Pacific Northwest populations possess intermediate genetic distances to East Asian populations (mean pairwise *F*_ST_ = 0.069 with SD = 0.011; Table S2), Central and South American populations (mean pairwise *F*_ST_ = 0.112 with SD = 0.031; Table S2), and populations of Europe, the Middle East, and Central and South Asia (mean pairwise *F*_ST_ = 0.068 with SD = 0.024; Table S2), with the larger value in the comparison to other Native Americans likely reflecting the inflation of *F*_ST_ in computations involving populations with lower heterozygosities [33] rather than a particularly strong genetic difference. Indeed, our *F*_ST_-based multidimensional scaling (MDS; Figure 3A) and neighbor-joining (Figure 3B) analyses of the 71 populations in the combined dataset place the Pacific Northwest populations intermediate among the East Asian populations, Central and South American populations, and populations of Europe, the Middle East, and Central and South Asia, with the closest connection observed to Central and South Americans.

In general, the shared ancestry identified in the population-level MDS and neighbor-joining plots accords with geography, in agreement with previous observations [28], [29], [34], [35]. African and South American populations cluster far apart (Figure 3), in accord with high *F*_ST_ values between these groups (mean pairwise *F*_ST_ = 0.281 compared to a 95% quantile of 0.222 across all comparisons; SD = 0.047 across 30 pairs involving 10 African and three South American populations; Table S2). Similarly, comparisons between South American and Oceanian populations have high *F*_ST_ values (mean pairwise *F*_ST_ = 0.277 with SD = 0.031 across six pairs), and these populations cluster far apart in MDS and neighbor-joining. In the MDS plot (Figure 3A), Middle Eastern, European, and Central and South Asian populations form a central cluster (mean pairwise *F*_ST_ = 0.022 with SD = 0.014 across 276 pairs), separate from a tight cluster containing the 21 East Asian populations (mean pairwise *F*_ST_ = 0.013 with SD = 0.010 across 210 pairs). Considering all comparisons between East Asian populations and Middle Eastern, European, and Central and South Asian populations, mean pairwise *F*_ST_ is 0.091 (SD = 0.024, 504 pairs). The African American and Mexican American populations cluster at intermediate distances between the European cluster and most African and Central American populations, respectively, reflecting the expectation of intermediate placement for admixed groups [9], [36]–[39]. Note that due to differences in their dimensionality reduction approaches, the MDS and neighbor-joining analyses are not always in agreement: for example, the Seri and Pima from northwestern Mexico cluster together on the MDS plot, whereas they have a relatively high *F*_ST_ value (0.094), as shown by the long branches separating these populations on the neighbor-joining tree.

### Individual-level analysis: Complex admixture patterns in the Americas

#### Genomic differentiation worldwide

**Figure 4:**
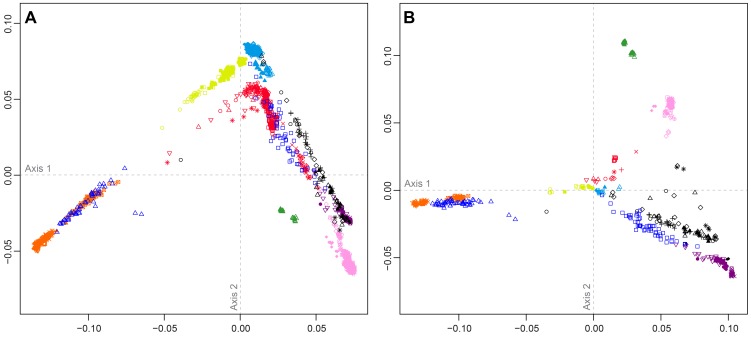
Individual-level population structure. (A) Multidimensional scaling plot of pairwise allele-sharing distance (ASD) among 2,140 individuals in the combined dataset. (B) Multidimensional scaling plot of pairwise ASD among 528 individuals from 63 worldwide populations, following the resampling of a maximum of 82 individuals each from 11 different population groups. Group choices for resampling were taken from Figure S6. Population colors and symbols follow Figure 1.

Figure 4 shows MDS analyses based on pairwise allele-sharing distances (ASD) among individuals in the combined dataset. As has been seen in previous studies [29], [34], [35], [40], high allele-sharing distances between Africans and East Asians determine one of the two first dimensions (Figure 4A), while distances between Europeans and East Asians determine the other. Mexican Americans appear near Central and South Asians, along an axis connecting Europeans and East Asians. Indigenous Northwest individuals lie along a distinct parallel axis, connecting Europeans to a cluster of South Americans that is close to, but separate from, the East Asian cluster. Three Indigenous Northwest individuals cluster with Europeans, potentially indicating recent European admixture among the Pacific Northwest populations that was not evident in our population-level analyses.

The individual-level MDS plot in Figure 4A differs somewhat from the population-level MDS plot in Figure 3A, as Euclidean distances between centroids of the individuals within populations in Figure 4A are imperfectly correlated with corresponding Euclidean distances between populations in Figure 3A (Spearman *ρ* = 0.655, *P* = 0.002). This apparent discrepancy potentially arises from uneven sample sizes that can mask differences between geographic regions represented by fewer individuals. Thus, for example, while Oceanians and South Americans have high genetic differentiation (mean ASD = 0.301 with SD = 0.004 among 784 individual pairwise comparisons), these populations have less influence on the plot than more numerous Europeans and East Asians, who have slightly lower ASD (mean ASD = 0.298 with SD = 0.003 among 216,450 comparisons) but are placed much farther apart in Figure 4A.

To reduce the effect of uneven sampling on the MDS analysis, we randomly subsampled individuals based on their worldwide geographic distribution (see Materials and Methods). In an MDS analysis of a reduced dataset with 11 worldwide groups of populations, each with a maximal sample size of 82 individuals—the number of Pacific Northwest individuals in our combined dataset—we instead find the Euclidean distances between centroids of individuals within populations (Figure 4B) to have greater agreement with population placements in the population-level analysis of Figure 3A (*ρ* = 0.938, *P*<2.2×10^−16^).

The subsampled plot further clarifies relationships in the Americas. Unlike in Figure 4A, in which Mexican Americans are superimposed on Central and South Asians, in Figure 4B, they are distributed between the source regions of Europe and Central and South America. Central and South Americans cluster separately from Pacific Northwest individuals, who are placed between three clusters: Central and South Americans, East Asians, and European, Middle Eastern, and Central and South Asian populations. Two Tlingit individuals and one Stswecem'c individual cluster with Europeans, possibly reflecting European admixture. Two Splatsin individuals and one Tlingit individual lie at an intermediate distance between the Central and South American and East Asian clusters, while a single Haida individual lies between the American, European, and African clusters. Thus, the analysis of subsamples illuminates the distinctiveness of admixture histories among Pacific Northwest, Mexican American, and Central and South American individuals. While Mexican Americans and some Mayans and Colombians show signals mainly of European contributions in addition to the indigenous component, Pacific Northwest individuals appear to possess recent admixture with both European and East Asian sources, and for one individual, with Africans.

#### Genomic differentiation among Eurasian and American populations

We performed additional MDS analyses to further investigate genomic clustering patterns for the Pacific Northwest, restricting our attention to randomly sampled subsets of individuals from Eurasia and the Americas (see Materials and Methods). These analyses assessed the alignment of the Pacific Northwest individuals on paths in MDS plots connecting potential source populations.

**Figure 5:**
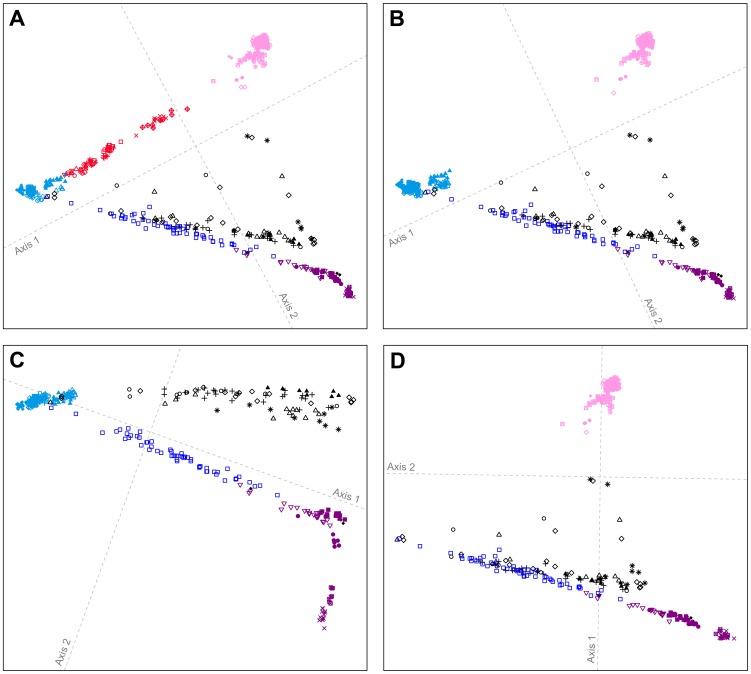
Procrustes-transformed multidimensional scaling plots of Eurasian and American individuals. (A) 641 individuals from 53 populations after resampling of 82 individuals from each of 14 nonoverlapping groups of European, Central and South Asian, East Asian, and American populations (Figure S7). (B) 641 individuals from 41 populations after resampling of 82 individuals from each of 15 nonoverlapping groups of European, East Asian, and American populations (Figure S8). (C) 393 individuals from 22 populations after resampling of 82 individuals from each of 10 nonoverlapping groups of European and American populations (Figure S9). (D) 450 individuals from 34 populations after resampling of 82 individuals from each of 11 nonoverlapping groups of East Asian and American populations (Figure S10). Procrustes similarity statistics are *t*_0_ = 0.958 between Figures 4B and 5A, *t*_0_ = 0.999 between Figures 5A and 5B, *t*_0_ = 0.956 between Figures 5B and 5C, and *t*_0_ = 0.997 between Figures 5B and 5D. Population colors and symbols follow Figure 1.

Considering 641 individuals from Europe, Central and South Asia, East Asia, and the Americas, clustering patterns remain largely unchanged from those at the worldwide scale, with the Pacific Northwest individuals placed largely on paths toward the Europeans and East Asians (Figure 5A; Procrustes similarity statistic *t*_0_ = 0.958). Exclusion of Central and South Asians from the analysis leaves the locations of European, East Asian, and American individuals largely unchanged (Figure 5B; *t*_0_ = 0.999); because many of the Pacific Northwest individuals continue to lie on axes oriented toward the Europeans and East Asians, this result suggests that the Europeans and East Asians, and not the Central and South Asian populations, are more likely to be sources of recent admixture signals in the Pacific Northwest.

If we consider only individuals of European and American origin (Figure 5C), then the American individuals form three clusters: (i) South American Karitiana and Surui at the bottom right, (ii) Mexican American, Mayan, Pima, Seri, and Colombian individuals along the first dimension, and (iii) Pacific Northwest individuals distributed from left to right at the top of the plot. The alignment toward Europeans supports recent European admixture in the Mexican American, Mayan, and Pacific Northwest populations, but not in the Karitiana and Surui. If we consider only the East Asian and American individuals (Figure 5D), the clustering patterns are similar to Figure 5B (*t*_0_ = 0.997), with some Pacific Northwest individuals oriented toward East Asians. This result provides support for recent East Asian admixture in some Pacific Northwest individuals, but not in other Native Americans.

#### Genomic differentiation among Pacific Northwest populations

**Figure 6:**
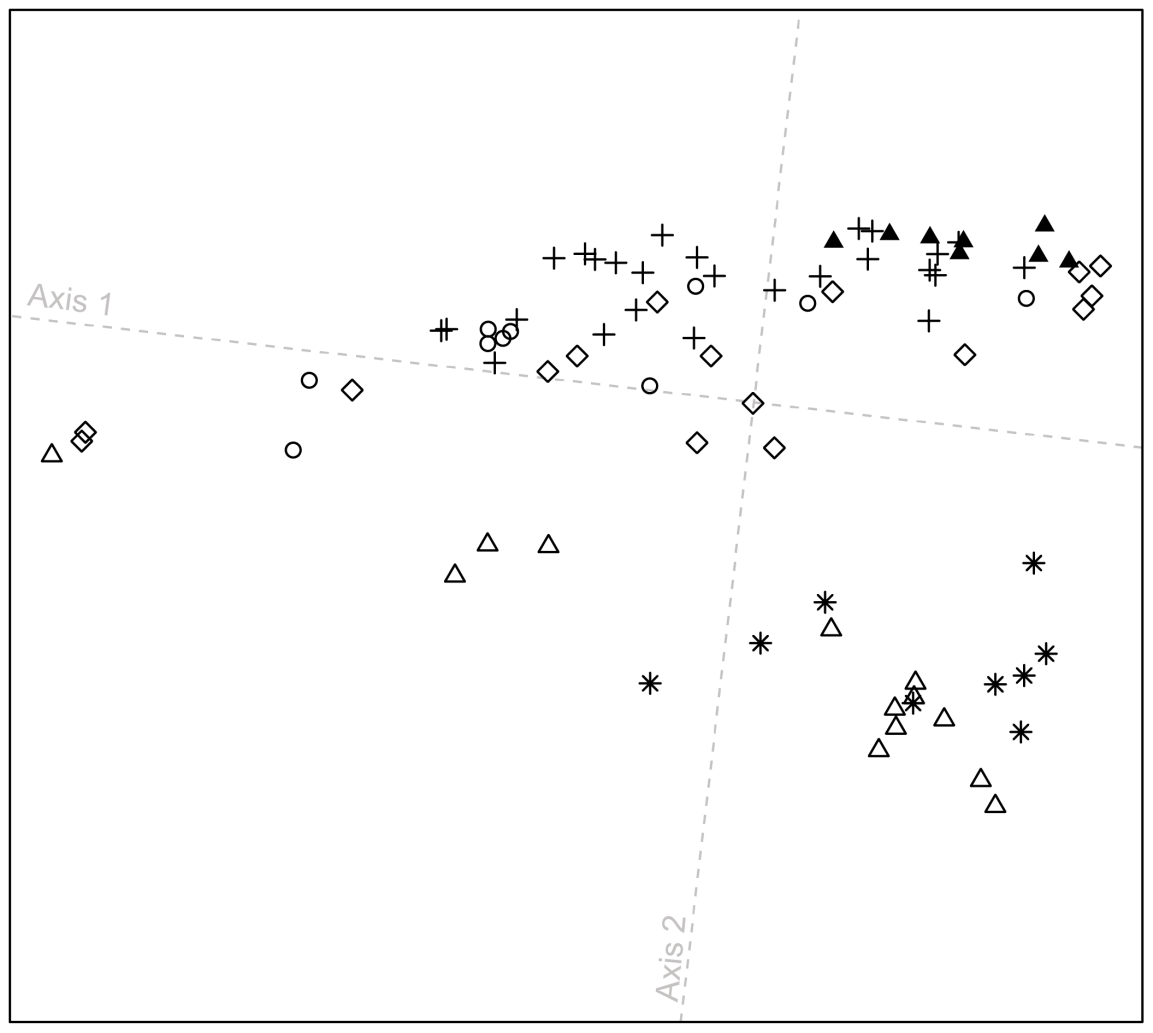
Procrustes-transformed multidimensional scaling plot of Pacific Northwest individuals. The plot is based on pairwise ASD among 82 individuals from six indigenous populations; *t*_0_ = 0.874 between overlapping individuals in Figures 5C and 6. Population colors and symbols follow Figure 1.

At a local geographic scale in the Pacific Northwest, in a plot excluding all other populations (Figure 6), the distribution of individuals along the first dimension is broadly similar to the patterns observed in Figure 5. Furthermore, the figure highlights that most Splatsin and Stswecem'c individuals cluster separately from other indigenous Pacific Northwest individuals (average linkage distance *L*_0_ = 0.028, with SD = 0.009, between Splatsin and Stswecem'c individuals, and all other Pacific Northwest individuals; *P*<0.001). These two populations originate from interior British Columbia, whereas the other four populations are coastal, suggesting a difference in demographic history for interior and coastal Pacific Northwest groups. The pattern is consistent with coastal and interior populations being placed in different cultural regions as defined by the Smithsonian Handbook of North American Indians [41], supporting the distinction between coastal and interior groups in our study design.

#### Admixture at the worldwide scale

**Figure 7:**
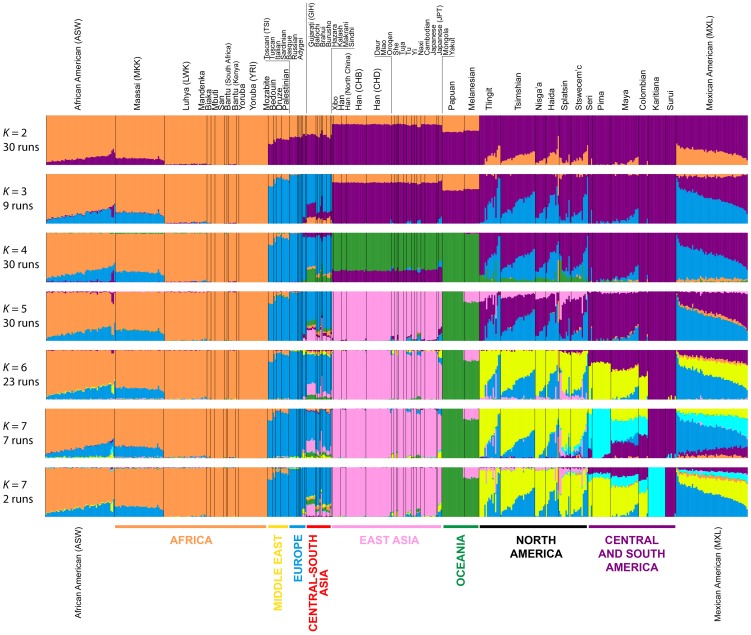
Worldwide Admixture structure. Plotted are modes with clustering solutions obtained with 30 replicates at each value of *K*. Values of *K* and the number of runs in the mode shown appear on the left. In each plot, each cluster is represented by a different color, and each individual is represented by a vertical line divided into *K* colored segments with heights proportional to genotype memberships in the clusters. Thin black lines separate individuals from different populations. The same 528 individuals included in Figure 4B are considered in the Admixture analyses. Alternate clustering solutions for values of *K* from 2 to 12 appear in Figure S3.

To refine our perspective on admixture in Native Americans suggested by MDS, we used the model-based software Admixture [42] on the same sets of individuals from our worldwide analyses (Figure 4B) and our analyses restricted to European, East Asian, and American populations (Figure 5B). Because our emphasis is on the Pacific Northwest, we focus on the clustering solutions for values of *K* from 2 to 7, which identified clusters specific to the Native American populations (Figure 7). Clustering solutions for other values of *K* from 2 to 12 appear in Figures S3 and S4.

The Admixture patterns observed for global populations provide a basis for interpreting the placement of the Pacific Northwest populations. Using the same set of 528 worldwide individuals included in Figure 4B, at *K* = 2, West Africans possess ∼100% membership in the orange cluster, and South American Karitiana and Surui individuals have 100% membership in the purple cluster (Figure 7). At *K* = 3, Europeans land mainly in the new blue cluster, with ∼100% membership, and Middle Eastern and Central and South Asian individuals largely resemble Europeans (mean memberships in the blue cluster = 0.859 with SD = 0.071 and mean = 0.679 with SD = 0.105, respectively). Unlike most Central and South Americans, at *K* = 3, most Pacific Northwest individuals resemble admixed Mexican Americans in their membership profiles (mean membership in the blue cluster = 0.344 with SD = 0.226 and mean = 0.514with SD = 0.150 respectively). One exception is that the Haida individual located near Africans in Figure 4B has greater membership in the orange cluster (0.436) than all other individuals in this population (mean = 0.007 with SD = 0.017). The Central or South American population with the most similar membership profile to the Pacific Northwest is the Mayans; while individuals in this population have substantial membership in the purple cluster, they also have high coefficients for the blue cluster suggestive of European admixture (mean = 0.112 with SD = 0.090).

At *K* = 4, all 30 runs of Admixture produce a new green cluster for Oceanians, who have ∼100% membership in this cluster, and for East Asians, who have majority membership. A membership signal for this cluster is visible in the Pacific Northwest individuals, but not in Central and South Americans. At *K* = 5, the new pink cluster is most pronounced in the East Asians; in a similar manner to the pattern at *K* = 4, while Pacific Northwest individuals have substantial membership in the pink cluster at *K* = 5 (mean = 0.140 with SD = 0.113), Central and South American populations do not (mean = 0.023 with SD = 0.012). At *K* = 6, the new yellow cluster is centered on the Pacific Northwest (mean = 0.627 with SD = 0.214) and the Seri, Pima and Mayan (mean = 0.615 with SD = 0.115) populations. Pacific Northwest individuals no longer have appreciable membership in the purple cluster, whereas Central American and Colombian individuals retain substantial membership in this cluster (mean = 0.356, SD = 0.097). Because the yellow cluster subsumes mostly the formerly purple membership in the Pacific Northwest populations and, to a lesser extent, some of the pink but not the blue component from *K* = 5, this cluster likely represents Native American ancestry components distinct from those of Central and South Americans, tracing to genetic differentiation that originated prior to admixture events that followed European contact. Further support for this hypothesis is provided at *K* = 7 and higher, for which clustering solutions for Pacific Northwest populations do not change appreciably from those at *K* = 6 (Figures 7 and S3). Instead, two clustering solutions at *K* = 7 differ from the *K* = 6 pattern primarily in the Central and South American populations, reflecting additional subdivision among those groups (Figure 7).

#### Admixture for Eurasian and American populations

**Figure 8:**
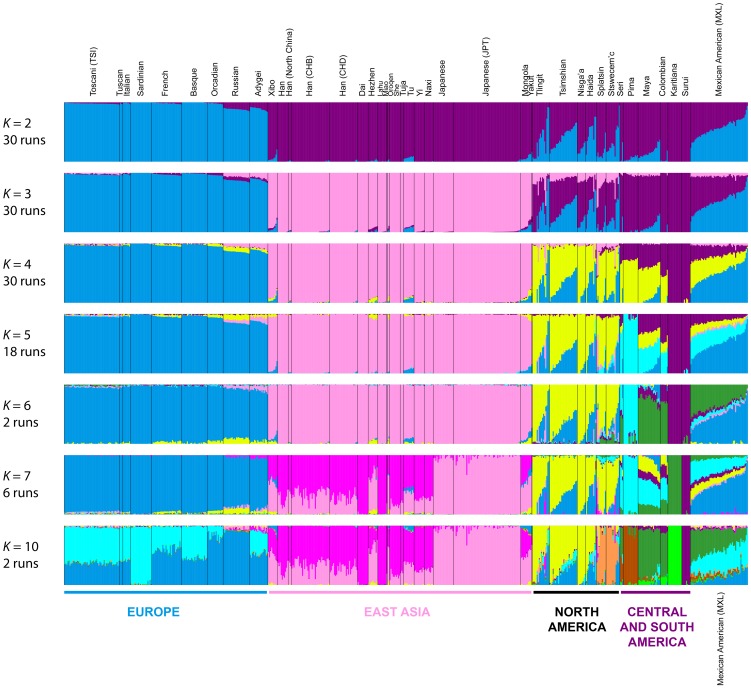
Admixture structure in the Americas. Plots are as described in Figure 7. The same 641 individuals from 41 European, East Asian, and American populations included in Figure 5B are considered in the Admixture analyses. Alternate clustering solutions for values of *K* from 2 to 12 appear in Figures S4.

If we consider the same 641 East Asian, European, and American individuals included in Figure 5B, at *K* = 2, Europeans possess ∼100% membership in the blue cluster and South Americans have ∼100% membership in the purple cluster (Figure 8). At *K* = 3, the new pink cluster is centered on East Asians, and Northern and Central American populations have substantial membership in each of the three clusters. At *K* = 4, the new yellow cluster has greatest representation in Northern and Central Americans, consistent with the worldwide analysis at *K* = 6 (Figure 7). At *K* = 5, the new light-blue cluster emerges predominantly in Central American Seri, Pima and Mayan individuals, as in one of the clustering solutions in the *K* = 7 worldwide analysis (Figure 7).

The new green cluster at *K* = 6 is visible primarily in the Seri, Mayan, Colombian and Mexican American individuals (Figure 8); this cluster appears only at *K* = 11 and *K* = 12 in the worldwide analysis (Figure S3). An alternative clustering solution for this green cluster emerges at *K* = 7, where Karitiana individuals now have ∼100% membership in the green cluster otherwise present to a much smaller extent in Mayans and Colombians (Figure 8 and S4).

Although no further clustering solutions specific to indigenous American populations emerge at *K* = 8 and *K* = 9 (Figure S4), we do observe two runs at *K* = 10 in which a new orange cluster arises almost exclusively in the interior Splatsin and Stswecem'c Pacific Northwest individuals (Figure 8), replacing their previous membership in the yellow cluster. The emergence of this cluster echoes the distinct genomic patterns between coastal and interior Pacific Northwest populations observed in the MDS analyses (Figure 6).

#### Time since admixture in Northwest and Central America

To investigate the time of admixture of Native American populations with Europeans and East Asians, we estimated the mean most recent time of admixture compatible with observed admixture distributions among individuals within each population, using the single-historical-event admixture model in Verdu and Rosenberg [43] together with membership proportions from the Admixture analyses in Figure 8. This computation is conditional on a simple model, and while its estimates are a first approximation, even if they imprecisely reflect absolute times of onset of admixture, differences in admixture time estimates can be informative about differences in the admixture histories experienced by the various populations.

Using this approach, separately for each population, we estimated a mean most recent time of European admixture that is consistent with the population's mean and variance of individual European admixture levels. We obtained an estimate of 78 years before present (YBP; SD = 4 years on average across values of *K*) for coastal Pacific Northwest populations (Tlingit, Tsimshian, Nisga'a and Haida) and 63 YBP for the interior Splatsin and Stswecem'c populations (SD = 7, across values of *K*). Furthermore, we obtained older estimates for the Central American Mayan and Mexican American populations (mean = 108 YBP with SD = 12, and 112 YBP with SD = 2, respectively). Finally, we find evidence for a slightly older East Asian admixture event in coastal compared with interior Pacific Northwest populations (mean = 90 YBP with SD = 23, and mean = 80 YBP with SD = 12, respectively). However, this latter result should be regarded with caution given the larger variance among estimates across values of *K*, likely due to difficulties in estimation of small absolute levels of East Asian admixture in our sample set (Figure 8).

We further estimated population-specific times of onset of European or East Asian admixture using the admixture linkage disequilibrium approach implemented in the software package *ALDER* [44]. For European admixture (Table 2), we obtained time of admixture estimates that are slightly older but in qualitative agreement with those obtained with the approach of Verdu and Rosenberg [43]. We obtained an estimate of the onset of European admixture of 101 YBP (mean SD = 13, with the mean taken across admixture time standard deviations) on average among coastal Pacific Northwest populations, and 127 YBP (mean SD = 20, with the mean taken across admixture time standard deviations) on average among interior populations (Table 2). Consistent with the Verdu and Rosenberg [43] method, we also found older admixture onset times for European admixture in the Mexican American and Maya populations (197 YBP with average SD = 9, 177 YBP with average SD = 26).

**Table 2:** Time of European and East-Asian admixture in North and Central America estimated using the admixture linkage disequilibrium approach in *ALDER* [44].

*North and Central America* α	*European admixture* β	Mean admixture time ± mean SDδ	Mean admixture rate ± SD∈
Tsimshian	*EurA*	**94±7**	28.80±3.90
Haida	*EurA*	**100±15**	31.63±5.22
Nisga'a	*EurA*	**106±13**	9.40±0.93
Tlingit	*EurB*	**106±16**	24.73±3.39
Stswecem'c	*EurC*	**111±16**	25.94±3.15
Splatsin	*EurA*	**142±24**	10.93±0.12
Mexican American	*EurC*	**197±9**	44.76±4.78
Maya	*EurA*	**177±26**	9.83±0.79

*North and Central America* α	*East Asian admixture* γ		
Tsimshian	*na*	*na*	*na*
Haida	*AsA*	**71±33**	2.65±0.07
Nisga'a	*na*	*na*	*na*
Tlingit	*AsB*	**89±21**	3.92±0.08
Stswecem'c	*AsB*	**92±30**	4.30±0.23
Splatsin	*AsC*	**209±55**	12.40±0.04
Mexican American	*na*	*na*	*na*
Maya	*na*	*na*	*na*

α Populations considered as admixed populations using *ALDER* [44].

β Sets of European populations considered separately in *ALDER* [44] as reference populations for admixture.

*EurA* : Toscani (TSI); Caucasian (CEU); Russian; Basque; French; Sardinian.

*EurB* : Toscani (TSI); Basque ; French; Sardinian.

*EurC* : Toscani (TSI); Caucasian (CEU); Basque; French; Sardinian.

γ *Sets of* East Asian populations considered separately in *ALDER* [44] as reference populations for admixture.

*na*: No significant admixture was found with any of the reference populations considered.

*AsA* : Japanese (JPT); Japanese.

*AsB* : Han; Han (CHB); Han (CHD); Japanese (JPT); Japanese.

*AsC* : Han; Han (CHB); Han (CHD); Japanese (JPT); Japanese; Yakut.

δ Mean admixture time in years (25 years for generation time) estimated by *ALDER* [44] across the reference populations considered ± mean of the admixture time standard deviations obtained across the reference populations considered.

∈ Mean admixture rate estimated by *ALDER* [*44*] across the reference populations considered ± standard deviation.

Using *ALDER* [44], we found (Table 2) significant traces of small absolute levels of East Asian admixture only in the coastal Haida (mean East Asian admixture level 2.65%, SD = 0.07 across East Asian reference source populations) and Tlingit (mean East Asian admixture level 3.92%, SD = 0.08 across East Asian reference source populations) populations, and in the interior Splatsin (mean East Asian admixture level 4.30%, SD = 0.23) and Stswecem'c populations (mean East Asian admixture level 12.40%, SD = 0.04). These admixture events were estimated to have occurred on average 80 YBP (mean SD = 27 with the mean taken across admixture time standard deviations) among the two coastal populations and 150 YBP (mean SD = 42) in the two interior populations. The relatively wide confidence intervals for the East Asian admixture onset times are likely due to uncertainty in estimates when East Asian admixture levels are small overall, and thus, should be regarded with caution.

Taken together, these results provide evidence of differences in the admixture histories for coastal and interior Pacific Northwest populations as well as with Central American Mayan and Mexican American populations, consistent with the patterns observed in our MDS (Figures 4 and 5) and Admixture analyses (Figures 7 and 8).

## Discussion

Previous investigations in multiple fields have proposed that populations originally from Asia migrated into the Americas via Beringia after the last glacial maximum and subsequently colonized the continent via north–south migration [1], [4], [13], [17], [45]–[48]. The origin and number of migration waves into the Americas, the pre-contact demography of the populations, and the post-contact recent history of admixture after European contact all represent topics of great interest for understanding the population history of these continents [4], [13], [17]. Despite this interest, however, relatively few genomic investigations of indigenous North American populations have been conducted [13], [48], and most have been centered on Central and South American groups [15], [18]–[21], [23].

To address this imbalance, with an interest in post-contact admixture, we investigated genome-wide SNP diversity in six Pacific Northwest populations, representing coastal and interior regions previously proposed to lie along separate migratory routes from Beringia [4]. The results provide insight into features of migration and admixture in the Pacific Northwest region, as well as differences in population-genetic history from the more frequently studied populations of Central and South America.

### Shared ancestry for Northwest North America

Various analyses of population structure placed the Pacific Northwest populations in relatively close genetic proximity, suggesting that these populations share an indigenous component of ancestry more recent than their divergence from other groups. Native American populations were distributed from north to south along a single branch of the neighbor-joining tree, as would be expected under a scenario with a common origin for all of the Native American groups followed by a north–south serial-founder model [16], [28]–[32], [34]. Hierarchical genetic structure among Native American populations detected using Admixture identified clusters specific to Northern, Central, and Southern indigenous American populations within a broader cluster comprising all Native Americans, as might be expected under the model.

In the Pacific Northwest, both our MDS analysis and an Admixture cluster at *K* = 10 revealed substantial genetic differentiation between coastal and interior populations. It is perhaps plausible that these population groups descend from different groups along separate migratory routes from Beringia into North America [4], [48]. However, because both population groups clustered together consistently in all other Admixture analyses, and they are placed nearby in MDS plots and in the neighbor-joining analysis, our results provide stronger support for a shared origin for the Pacific Northwest populations, and, after the initial peopling of the region, divergence due to isolation and drift. This scenario is consistent with paleoanthropometric studies that also proposed recent isolation, drift, and ecological differences to explain skeletal differences between coastal and interior individuals in British Columbia [49], [50].

Genetic diversities among Pacific Northwest populations were higher than expected under a serial-founder model, as the model predicts intermediate levels of diversity between Northeast Asians and Central and South Americans [31], [32]. Instead, however, heterozygosity levels among Pacific Northwest populations are substantially closer to those of Eurasian populations than to those of Central or South Americans. This result parallels the patterns observed in African Americans and Mexican Americans, two recently European-admixed populations in the Americas, who also showed inflated levels of genetic diversity compared to African and Native American source populations, respectively [9], [29], [34]. It is thus possible that admixture events following European contact might explain high genomic diversity in the Pacific Northwest populations in relation to Central and South Americans [16], [24], [25].

### Delayed history of European admixture in Pacific Northwest compared to Latin America

Our MDS and Admixture analyses produced high mean levels of European admixture in Pacific Northwest populations compared with Native American populations from Central and South America. Indeed, we observed high levels of European admixture in the Tlingit, Tsimshian and Haida populations comparable in magnitude to the recently admixed Mexican American population. This result contrasts with patterns in the Amazonian Karitiana and Surui populations, for which no admixture signals were evident, and with the low levels of European admixture observed in Colombians and Central American groups [13], [18]–[21], [23].

Our estimates of the most recent time of admixture support a longer history of European admixture among Central American admixed populations than among Pacific Northwest populations, with the within-population variance of individual admixture estimates across individuals higher in the Pacific Northwest. This result accords with the delayed post-European contact admixture processes in the Pacific Northwest relative to Central and South America [24], the later arrival of Russian and Northern European migrants in the Pacific Northwest fur trade toward the end of the 1700s, and the later colonization period centered on fishing and canning [25], relative to the Spanish and Portuguese colonial periods beginning after 1492.

### Recent East Asian admixture unique to Northwest North America

We detected signals of East Asian admixture in several Pacific Northwest populations, particularly the interior Splatsin and Stswecem'c groups. Consistent with previous studies, we observed no signal of genome-wide East Asian admixture in our set of Central and South American populations [28], [29], [34], [51]. It is possible that the East Asian admixture signal in the Pacific Northwest could represent waves of ancient Asian migrations into the Americas prior to European contact, or an inability of Admixture to fully separate genetic signals from similar groups. However, two features of the pattern support the view that it represents recent East Asian admixture. First, high variance in East Asian admixture proportions across individuals within Pacific Northwest populations indicates a relatively short and recent history of East Asian admixture, a pattern uniquely observed in this region of the Americas. Second, the pattern differs noticeably between the coastal and interior groups, two sets of populations that are otherwise difficult to distinguish. Thus, we surmise that the evidence for East Asian admixture reflects the documented history of Chinese and Japanese immigrants to British Columbia working in the mining, railway and cannery industries in the second half of the 19^th^ century [52], and that these groups had different influences on the coast and in the interior [53].

While our approach using two different methods [43] has provided simple strategies for estimating admixture times, the complexity of the admixture pattern in the Pacific Northwest, likely involving both European and East Asian sources and a different pattern in coastal and interior groups, suggests that simple models may be somewhat limited in applicability to the region. Future theoretical development of admixture models—that, for example, explicitly formulate pre- and post-contact admixture periods—together with approximate Bayesian computation or other techniques that can more fully incorporate admixture patterns into inference of the mechanistic admixture model, will help to enhance understanding of the variable histories of admixture experienced by indigenous American populations, both in the understudied Pacific Northwest and throughout the hemisphere.

## Materials and Methods

### Population samples and ethics statement

We collected DNA samples for 101 individuals from six indigenous populations of British Columbia (Nisga'a *n* = 8; Splatsin *n* = 16, Stswecem'c *n* = 15; Tsimshian *n* = 32) and Southeastern Alaska (Haida *n* = 12; Tlingit *n* = 18), and for three Seri individuals from northwestern Mexico (Figure 1). Collection of the Haida and Tlingit samples was approved by the Institutional Review Board (IRB #10379) at Washington State University, as described by Villanea et al. [54]. Appropriate informed consent and sample collection protocols for the communities from British Columbia and Alaska was approved by Institutional Review Boards at the University of Illinois (IRB #10538). Each participant from British Columbia and Alaska provided familial anthropology information concerning geographic and tribal affiliation of their maternal and paternal lines. For all individuals and populations, knowledge of family histories, including possible recent admixture events, were obtained through classical familial anthropology interviews.

#### Community engagement

Following consultation from the tribal councils, community meetings or both, we collected saliva samples using DNAGenotek Saliva Sampling Kits or Norgen Biotek Corp. Saliva DNA Collection, Preservation and Isolation Kits. On a regular basis we visited the communities in British Columbia to provide the latest results of the research study and answer any questions asked by community members. We gave public presentations on the “uses and limitations of using DNA variation to study population history.” Lastly, some community members from British Columbia attended the Summer Internship for Native Americans in Genomics (SING) workshop, convened at the Institute for Genomic Biology at the University of Illinois Urbana-Champaign. This workshop provides training for Native Americans in genomic and bioinformatics techniques as well as opportunities to discuss ethical, legal and social implications of genomic research specific to indigenous communities.

### SNP genotyping and quality control

#### SNP genotyping

In each of the 104 individuals, we obtained genotypes at 616,794 SNP loci included on the Illumina 610-Quad genotyping array, using a minimum of 500 ng of DNA per individual and following the manufacturer's recommended protocol. Raw data generation used an Illumina BeadArray Reader with BeadScan Software, and genotypes were called using the Genotyping (GT) Module (v1.7.4) of Illumina GenomeStudio (v2010.2). We restrict our analyses to autosomal SNPs; consequently, the 26,507 CNV and 15,270 non-autosomal markers present on the array were excluded. We further excluded 992 SNPs with ambiguous genomic position and 1,981 SNPs that failed genotyping in the sample set (Stage 1, Figure S5). We therefore retained 572,044 autosomal SNPs for further analysis, representing 92.75% of the SNPs on the genotyping array.

#### Quality control

We applied a three-stage quality control procedure (Figure S5). First, we performed quality control at the genotype-calling level, after which the preliminary dataset contained 565,635 autosomal SNPs with genotypes in 101 individuals; we excluded 6,407 SNPs with a call rate below 90% or a cluster separation below 0.2, two SNPs found to be duplicates, and three individuals with more than 10% missing data (Stage 1, Figure S5). Next, we performed a population-genetic quality control following Pemberton et al. [40]. We excluded 19,940 SNPs monomorphic in the sample of 101 individuals (Stage 2, Figure S5). The relatively high number of monomorphic SNPs is consistent with an absence of Pacific Northwest populations in the ascertainment set for the SNPs on the genotyping array. The initial dataset contained 545,697 autosomal SNPs with genotypes in 101 individuals.

### Pairwise relatedness

The presence of related individuals in a dataset can influence genetic diversity patterns [40], [55]. We therefore identified pairs of close relatives in the initial dataset using identity-by-state (IBS) allele-sharing and the likelihood approach of Relpair (v2.0.1) [56], [57]. Following Pemberton et al. [40], Relpair was applied to five non-overlapping sets of 9,999 SNPs (the maximum number of markers allowed by Relpair) in which all SNPs were separated by at least 100 kb. In these analyses, we considered only the 210,639 autosomal SNPs that were polymorphic in all seven indigenous populations, using genetic map positions obtained by interpolation on the Rutgers combined physical–linkage map [58], [59]. We set all putative pairwise relationships to “unrelated,” the genotyping error rate to 0.001 (a likely overestimate), and the critical value for likelihood ratio computation to 100. We only considered first- and second-degree relationship inferences, as cousin inferences are less reliable than inferences for closer relationships [40], [56], [57]. To exclude intra-population relative pairs, separately in each population, we applied Relpair using count estimates of allele frequencies in that population. To exclude inter-population relative pairs, we applied Relpair to the whole dataset using count estimates of allele frequencies in the dataset.

We identified 24 intra-population first- and second-degree relative pairs that involved 36 distinct individuals: 11 in the Tsimshian population (four parent–offspring, two full-sibling, and five avuncular, involving 15 individuals in total), six in the Splatsin population (one parent–offspring, two full-sibling, and three half-sibling, involving eight individuals), three in the Stswecem'c population (one parent–offspring, one full-sibling, and one avuncular, involving five individuals), and two each in the Haida (two parent–offspring, involving four individuals) and Tlingit (one parent–offspring and one full-sibling, involving four individuals) populations. No inter-population pairs of close relatives were identified.

To minimize the number of individuals excluded, we first removed from the dataset 11 individuals that appeared in more than one pair. Next, we removed five individuals appearing in only a single pair, selected on the basis of higher levels of missing data. Following the removal of the 16 related individuals from the preliminary dataset containing 565,635 autosomal SNPs, we repeated the population-genetic quality control procedure (Stage 3, Figure S5) and excluded 20,914 SNPs monomorphic in the sample of 85 individuals and 339 SNPs with at least 10% missing data. Thus, our final dataset contained 544,384 autosomal SNPs with genotypes in 85 unrelated individuals from seven populations (Table 1). A version of this dataset restricted to the 82 unrelated individuals from six British Columbian and Alaskan populations newly sampled and genotyped here can be requested from R.S.M. for population and evolutionary history studies in accord with the informed consent documents used for this study.

### Merging with publicly available data

To investigate the indigenous populations in relation to genetic variation in other populations, we merged the indigenous Northwest dataset with similar publically available data for the 11 populations in release 3 of HapMap project [60] and the 53 populations represented in the HGDP-CEPH cell line panel. First, we separately prepared and merged the HapMap Phase III and HGDP-CEPH datasets using the pipeline of Pemberton et al. [61] and considering only autosomal SNPs; SNPs on the mitochondrion and on the X and Y chromosomes were excluded.

#### Preparation of HGDP-CEPH dataset

We considered 938 individuals from the H952 subset [55], in which no pair of individuals is more closely related than first cousins, for whom unphased genotypes at 644,258 autosomal SNPs were available [28] (downloaded June 26th, 2009). After quality control, the final data set contained 642,939 SNPs; 53 SNPs that were monomorphic, and 399 SNPs with >10% missing genotypes, in the 938 individuals were removed. A further 88 SNPs with fewer than five alleles in at least one population were omitted, and 779 SNPs were excluded because of Hardy-Weinberg disequilibrium.

#### Preparation of HapMap dataset

The HapMap Phase III data set [60] (release 3, downloaded September 9th, 2009) consisted of 1,397 individuals for whom unphased genotypes were available at 1,423,833 autosomal SNPs. We considered 1,117 unrelated individuals from the HAP1117 subset [40], in which no pair is more closely related than first cousins. After quality control, the final data set contained 1,405,599 SNPs; 424 SNPs that were monomorphic in the 1,117 individuals were removed, in addition to 17,810 SNPs excluded because of Hardy-Weinberg disequilibrium.

#### Merging of HGDP-CEPH and HapMap datasets

We assembled data on 2,055 individuals—938 HGDP-CEPH, 1,117 HapMap—from 64 populations and 590,461 SNPs shared by these two datasets. At 106,591 SNPs, the datasets had genotypes given for opposite strands, and we converted HGDP-CEPH genotypes to match the HapMap genotypes.

#### Preparation of combined dataset

We merged the combined HapMap and HGDP-CEPH dataset with the indigenous Northwest dataset at the 475,109 autosomal SNPs the datasets shared in common (Stage 4, Figure S5). At 90,832 SNPs, the indigenous Northwest dataset and combined HapMap and HGDP-CEPH dataset had genotypes given for opposite strands, and we converted the indigenous Northwest genotypes to match the combined HapMap and HGDP-CEPH genotypes. The final merged dataset (“combined dataset” henceforth) therefore contained 2,140 unrelated individuals from 71 worldwide populations with genotypes at 475,109 autosomal SNPs (Figure 1, Table S1).

### Population-genetic analyses at the population level

#### Haplotype heterozygosity

Use of haplotype statistics rather than per-SNP values provides a way of comparing heterozygosities with SNPs ascertained in geographically limited samples, and has been an informative approach for assessing population differences in heterozygosity for standard SNP panels such as those on Illumina arrays [28], [62]. Thus, in the combined dataset, we expanded the method of Li et al. [28] to the genome-wide scale, using combined recombination rate estimates from Phase II (Build 36.2) of the HapMap [60] to define blocks of high linkage disequilibrium (LD) in which to calculate SNP haplotype frequencies. Because the density of SNPs in the HapMap Phase II recombination map exceeded the number of SNPs in the combined dataset, for all intervals bounded by two contiguous SNPs in the combined dataset, we calculated the mean recombination rate across all HapMap SNPs within the interval. Next, we identified all blocks of 5–15 contiguous SNPs in the combined dataset in which all inter-SNP mean recombination rates were below 0.5 cM/Mb.

Using this approach, we defined a total of 11,408 high-LD SNP blocks, with a mean of 518.6 blocks per chromosome (SD = 282.2), ranging from 111 blocks on chromosome 22 (mean number of SNPs per block = 8.1, SD = 2.8) to 1,040 on chromosome 2 (mean number of SNPs per block = 7.8, SD = 2.7). The 11,408 blocks contained 87,246 of the 475,109 total SNPs, with a mean of 3,965 per chromosome (SD = 2,187).

Next, we calculated genome-wide mean haplotype heterozygosities in each population. Considering all individuals in a population, we estimated haplotype sample frequencies in each of the 11,408 haplotype blocks using the –hap-freq option in Plink [63], which constructs haplotypes in each block using an expectation-maximization phasing algorithm [64] prior to calculating their sample frequencies across individuals within the population. We then estimated expected haplotype heterozygosity for each block according to the Nei [65] formula, averaging these values across each chromosome for each of the 71 populations.

To evaluate how different block definitions might influence haplotype heterozygosities, we repeated the analysis using two alternative block definitions: (i) nonoverlapping blocks of 5 to 15 contiguous SNPs in which all inter-SNP mean recombination rates *exceeded* 0.5 cM/Mb, and (ii) 11,408 random non-overlapping blocks of 5 to 15 contiguous SNPs in the combined dataset. For both analyses, blocks were chosen such that their distribution of lengths exactly matched that of the blocks in the previous analysis with all inter-SNP mean recombination rates *below* 0.5 cM/Mb.

#### Distance from East Africa

To evaluate the relationship between haplotype heterozygosity and distance from Africa, we used the geographic coordinates of the seven new indigenous populations (Figure 1, Table1 and S1), taking the coordinates of the 64 HapMap Phase III and HGDP-CEPH populations from Pemberton et al. [61]. Next, we calculated each population's geographic distance from Addis Ababa, Ethiopia, along waypoint routes as defined in Ramachandran et al. [31] with *rdist.earth* from the *fields* package in R [66], using 6,371 km for the radius of the earth. Finally, we evaluated the linear regression between genome-wide mean haplotype heterozygosity and distance from Addis Ababa, providing the slope, the squared correlation coefficient (*R^2^*), and the Pearson product of moment correlation test *P* value obtained using cor.test in R. We excluded from this calculation three non-indigenous populations: African American (ASW), Caucasian (CEU), and Mexican American (MXL).

#### Population differentiation

We investigated genetic differentiation among the populations in the combined dataset using pairwise multilocus *F*_ST_ [67] calculated using 455,846 SNPs in the combined dataset with a minor allele frequency above 5% in the whole sample set. To visualize the 71×71 pairwise *F*_ST_ matrix, we first performed metric multidimensional scaling (MDS) using the *cmdscale* function in R. To evaluate how accurately the first two dimensions of the MDS analysis reflect the full 71×71 *F*_ST_ matrix, we calculated the Spearman rank-sum correlation *ρ* between the Euclidean distances separating all pairs of populations in the MDS plot and the corresponding *F*_ST_ values.

Second, we constructed a neighbor-joining tree (NJT) from the 71×71 pairwise *F*_ST_ matrix using the method of Gascuel [68], an improvement of the original algorithm of Saitou and Nei [69], using the *bionj* function from the package *ape* in R. To evaluate the robustness of the tree topology, we performed 1,000 bootstraps of the pairwise *F*_ST_ matrix and constructed 1,000 corresponding bootstrap trees. For each edge in the tree constructed from the true data, we counted how many of the trees constructed from the 1,000 bootstraps supported that edge.

### Population-genetic analyses at the individual level

#### Allele-sharing dissimilarities

We constructed a 2,140×2,140 allele-sharing dissimilarity (ASD) matrix between individuals with the program *asd* [70], using in the calculation for a given pair only those among the 475,109 SNPs for which both individuals had non-missing genotypes. Next, we performed MDS on this matrix using *cmdscale* in R. To evaluate the representation of the ASD matrix by the first two MDS dimensions, we calculated the Spearman rank-sum correlation *ρ* between Euclidean distances separating all pairs of individuals in the MDS plot and their corresponding ASD values.

#### Resampling within geographic groups

The combined dataset is heterogeneous both in terms of population sample sizes and in geographic representation (Figure 1, Table S1). Such heterogeneity might strongly influence the two-dimensional MDS projection of genetic dissimilarities among individuals. To address this issue, we created groups of populations based on geography, drawing random samples of equal size from each group to perform the MDS analysis. US Caucasians (CEU), who have ambiguous location of origin and genetically resemble other Europeans in our dataset, were excluded from these analyses.

In each analysis, we first created three population groups containing: (i) only British Columbian and Alaskan individuals, (ii) only African Americans (ASW), and (iii) only Mexican Americans (MXL). Next, we created a vector, *P*, containing all *I* remaining populations (equal to 62) in the combined dataset ordered by increasing geographic distance from Addis Ababa, and constructed a corresponding vector, *D*, containing the differences in geographic distance from Addis Ababa of contiguous populations in *P*, where *D*_j_ is the difference in geographic distance of populations *P*_j_ and *P*_j+1_ (*j* ∊ [1, *I*-1]). Considering all values in vector *D*, if *D*_j_ was less than a threshold distance *d*, we assigned populations *P*_j_ and *P*_j+1_ to the same population group; otherwise, they were assigned to different groups. Note that geographic groups defined this way can contain populations that are far from one another (Figures S6-c). We then randomly sampled without replacement 82 individuals—the total number of Indigenous Northwest individuals in the combined dataset—from each population group. In instances in which a population group contained fewer than 82 individuals, we included all available individuals in subsequent analyses. Finally, we applied MDS to a restriction of the pairwise ASD matrix containing only the sampled individuals.

We performed five different analyses. For each analysis, the sampling and MDS were repeated 1,000 times, and for each replicate, we computed the Spearman rank-sum correlation *ρ* between the Euclidean distances separating all pairs of individuals in the MDS plot and their corresponding ASD values. For each of the five analyses, we report the results corresponding to the replicate with highest *ρ* among the 1,000 replicates.

First, we performed an analysis at the worldwide scale, using *d* = 1,000 km to place the 62 populations into eight groups in addition to the Indigenous Northwest populations, the African Americans, and the Mexican Americans (Figure S6), with a total of 528 sampled individuals among the 2,140 total individuals in the combined dataset.

Next, we focused specifically on populations from Eurasia and the Americas, excluding the African Americans from the four subsequent analyses. We used *d* = 350 km, and performed four separate analyses that considered, in addition to the Indigenous Northwest and Mexican American groups: (i) only the 46 populations originating from Europe, Central-South Asia, East Asia, and the Americas, (ii) only the 36 populations from Europe, East Asia, and the Americas, (iii) only the 15 European and American populations, and (iv) only the 27 East Asian and American populations. In addition to the Indigenous Northwest and Mexican American groups, these strategies produced 12 (Figure S7), 13 (Figure S8), eight (Figure S9), and nine (Figure S10) population groups, respectively. These analyses considered 641, 641, 393 and 450 total sampled individuals, respectively.

#### Procrustes analyses

To formally compare different MDS analyses, we used a Procrustes approach [71]. For a given pair of two-dimensional MDS plots, this method transforms one plot to optimally align the positions of corresponding objects in the two plots. The Procrustes similarity statistic, 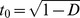, where *D* is the minimum sum of squared Euclidean distances between the two plots across all possible transformations, provides a formal measure of the similarity of the two plots after transformation (*t*_0_ ∊ [0,1], where *t*_0_ = 1 indicates an exact match of the two-dimensional positions of corresponding objects).

In the MDS plots of 82 individuals sampled from each population group, we performed four analyses that compared the two-dimensional positions of: (i) the 243 individuals shared in the worldwide MDS plot and the MDS plot restricted to Eurasian and American populations, (ii) the 450 individuals shared in the MDS plots restricted to Eurasian and American populations and restricted to European, East Asian, and American populations, (iii) the 287 individuals shared in the MDS plots restricted to European, East Asian, and American populations and restricted to European and American populations, and (iv) the 348 individuals shared in the MDS plots restricted to European, East Asian, and American populations and to East Asian and American populations.

Finally, we performed an MDS analysis based on pairwise ASD computed using all SNPs in the combined dataset and restricted to the six Pacific Northwest populations. We present the MDS plot after Procrustes transformation using as a reference the MDS plot restricted to European and American populations. We computed the average linkage distance between coastal and interior groups of individuals, *L*_0_ [72], [73], corresponding to the mean Euclidean distance on the two-dimensional MDS plot between two random individuals, one in the coastal group and the other in the interior group. We calculated the significance level of the observed clustering pattern between these two groups of individuals by computing a null distribution of *L*_0_ using 1,000 permutations of the “coastal” and “interior” labels among individuals in both groups. We report a *P*-value corresponding to the fraction of permuted *L*_0_ values greater than or equal to the observed *L*_0_ between coastal and interior groups.

#### Admixture

To investigate genetic structure in the combined dataset, we used the model-based clustering algorithm in Admixture v1.22 [42]. Admixture runs identify *K* genetic clusters, where *K* is specified by the user, without considering population affiliations of individuals. The method assigns each individual's genotype membership proportions in each cluster. Following author recommendations, prior to running Admixture, we LD-pruned the SNPs in each sample set, using the –indep-pairwise option in Plink [63] with a sliding window of 50 SNPs moved in increments of 10 SNPs, and an *r* ^2^ threshold of 0.1.

We performed two series of Admixture analyses, considering values of *K* from 2 to 12. First, we performed 30 independent runs of Admixture for each *K* using 67,158 LD-pruned SNPs in the set of 528 worldwide individuals that maximized the Spearman rank-sum correlation *ρ* between the Euclidean and ASD distances in the 1,000 replicate worldwide MDS analyses (see above). Second, we performed 30 independent runs of Admixture for each *K*, using 61,131 LD-pruned SNPs in the set of 641 individuals from 41 European, East Asian, and American populations that maximized the Spearman *ρ* between the Euclidean and ASD distances among the 1,000 replicates at the appropriate scale (see above). The geographically imprecise CEU population was excluded from the Admixture analyses.

For each Admixture run, the termination criterion was set to default following author recommendations: a run was terminated when log-likelihood increased by less than 10^−4^, regardless of the total number of iterations performed.

Separately for both series of analyses, we explored the clustering solutions inferred by Admixture across the 30 replicates for each value of *K* using the software Clumpp [74], which computes a symmetric similarity coefficient (SSC) between all pairs of Admixture runs at a given value of *K*. We defined runs with SSC>0.9 as belonging to the same Admixture clustering solution, or “mode,” and individual genotype membership proportion results were averaged across runs within modes prior to plotting using Distruct [75].

#### Time of onset of admixture using the method of Verdu and Rosenberg [43]

To further investigate differential genomic admixture patterns observed between coastal and interior Pacific Northwest populations, we computed an estimated time since admixture with populations of European and East Asian origin. We used eq. 26 from Verdu and Rosenberg [43], which estimates, considering a historical model with a single pulse of admixture, the time in generations since admixture as a function of the mean and variance of admixture fractions from a given source population in the hybrid population.

We calculated the moments of admixture fractions from a source population as the mean and variance of membership proportions in the corresponding Admixture cluster obtained using individuals of European, East Asian, and American origin. We considered European or East Asian admixture fractions as membership proportions in the blue or pink cluster, respectively, for *K* from 2 to 6 or 3 to 6 (at *K* = 2 the “pink” East Asian cluster does not exist), respectively, in all individuals from coastal Pacific Northwest populations (Tlingit, Tsimshian, Nisga'a and Haida), and separately in all interior individuals (Splatsin and Stswecem'c). For comparison, we applied the same approach to separately estimate the time since European admixture in the Mexican American (MXL) and Maya populations.

Note that eq. 26 in [43] provides an admixture time estimate in generations before the birth of individuals in the hybrid population. Therefore, we provide all results in years before present (YBP) by adding one generation (that of the sampled individuals) to the results obtained from eq. 26, considering a generation time of 25 years.

#### Time of onset of admixture using ALDER [44]

We investigated the time of onset of European or East Asian admixture separately by population, using the admixture linkage-disequilibrium approach implemented in *ALDER* [44]. For a given pair of admixed and reference (source) populations, *ALDER* estimates the time of admixture onset from the decay in admixture LD observed in the admixed population, fitted to the admixture LD decay theoretically expected under a model with a single pulse of admixture [44], [76], [77].

We lack non-admixed North and Central Native American source populations, and therefore considered the *ALDER* one-reference population model, separately using each Pacific Northwest population, the Mexican American population, and the Maya population as the admixed group. We considered only populations of European and East Asian origin with 20 or more individuals in our sample set as possible reference populations for the admixture events. We considered all 475,109 autosomal SNPs in our final data set for this analysis.

We then used *ALDER* to test, based on a weighted LD-based test analogous to the admixture *f*-tests from Reich et al. [78] and Patterson et al. [77], for the possible occurrence of admixture between each putative admixed population and European (Toscani (TSI), Caucasian (CEU), Russian, Basque, French, Sardinian) or East Asian (Han, Han (CHB), Han (CHD), Japanese (JPT), Japanese, Yakut) populations separately.

For all pairs of populations with significant levels of admixture identified, we used *ALDER* to estimate, separately for each admixed and reference pair, the time of onset of admixture and the mean admixture level in the admixed population, using default parameters recommended by the authors [44]. We provide in Table 2 the estimated time of admixture (generation time of 25 years) averaged across reference populations with which significant admixture was detected, the mean of the admixture time standard deviations obtained across reference populations, the mean admixture level estimated across reference populations, and the standard deviation of the mean admixture level across reference populations.

## Supporting Information

Figure S1 Genome-wide haplotype heterozygosities using haplotype blocks constructed from low-LD SNPs only. We define genome-wide haplotypes as nonoverlapping blocks of 5 to 15 contiguous SNPs in which all inter-SNP mean recombination rates are *above* 0.5 cM/Mb. (A) Mean expected haplotype heterozygosity in each population, with standard deviations across the 22 autosomes. (B) The correlation between mean haplotype heterozygosity and geographic distance from Addis Ababa.
(TIF)

Figure S2 Genome-wide haplotype heterozygosities using haplotype blocks constructed from random SNPs. We define genome-wide haplotypes as random nonoverlapping blocks of 5 to 15 contiguous SNPs in the combined dataset, each block containing the same number of SNPs as the blocks defined previously with all inter-SNP mean recombination rates *below* 0.5 cM/Mb (with a one-to-one correspondence between blocks). (A) Mean expected haplotype heterozygosity in each population, with standard deviations across the 22 autosomes. (B) The correlation between mean haplotype heterozygosity and geographic distance from Addis Ababa.
(TIF)

Figure S3 Alternative Admixture structure among worldwide populations for values of *K* from 2 to 12. Plots are described in Figure 7.
(TIF)

Figure S4 Alternative Admixture structure among European, East Asian, and American populations for values of *K* from 2 to 12. Plots are described in Figure 7.
(TIF)

Figure S5 Summary of quality control procedures.
(TIF)

Figure S6 Map of the population groups for analysis worldwide, used in Figure 4B.
(TIF)

Figure S7 Map of the population groups for analysis with the Eurasian and American populations, used in Figure 5A.
(TIF)

Figure S8 Map of the population groups for analysis with the European, East Asian, and American populations, used in Figure 5B.
(TIF)

Figure S9 Map of the population groups for analysis with the European and American populations, used in Figure 5C.
(TIF)

Figure S10 Map of the population groups for analysis with the East Asian and American populations, used in Figure 5D.
(TIF)

Table S1 Populations in the combined dataset. ^a^HapMap Phase III. ^b^HGDP-CEPH. ^c^This study. ^d^The distance to Addis Ababa along waypoint routes. ^e^Genome-wide mean haplotype heterozygosity and standard deviation across 22 chromosomes. ^f^The fraction of missing genotype data among the 475,109 total SNPs in the combined dataset, with the standard deviation taken across individuals within the population.
(DOCX)

Table S2 Matrix of pairwise genetic dissimilarities among populations estimated using pairwise *F*_ST_ [67].
(TXT)
