# Supplementary figures and images for: Patterns of Admixture and Population Structure in Native Populations of Northwest North America
Source: PLoS Genet. 2014 Aug 14;10(8):e1004530. doi: 10.1371/journal.pgen.1004530 (PMC4133047; doi:10.1371/journal.pgen.1004530)

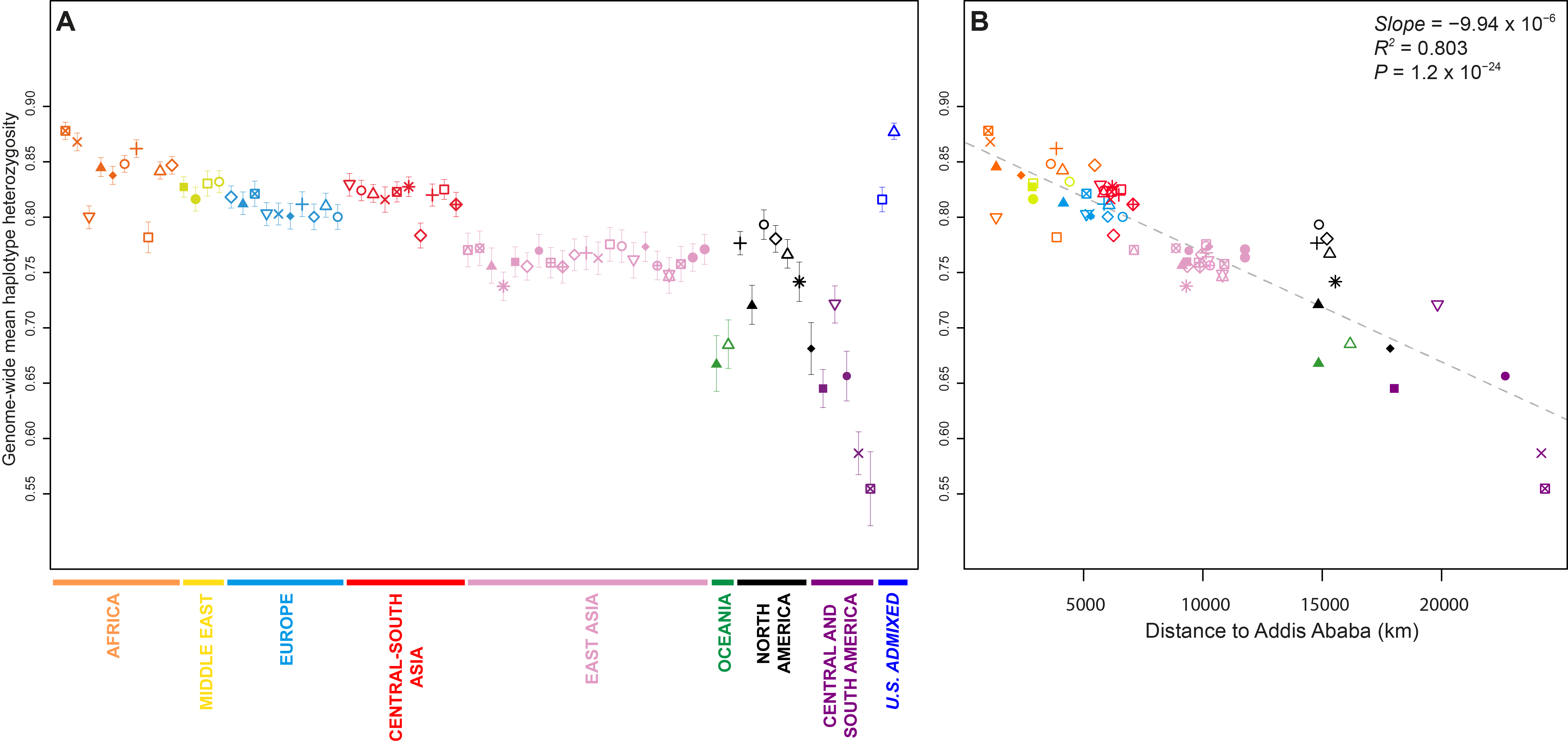

Supplement: Figure S1 — Genome-wide haplotype heterozygosities using haplotype blocks constructed from low-LD SNPs only. We define genome-wide haplotypes as nonoverlapping blocks of 5 to 15 contiguous SNPs in which all inter-SNP mean recombination rates are above 0.5 cM/Mb. (A) Mean expected haplotype heterozygosity in each population, with standard deviations across the 22 autosomes. (B) The correlation between mean haplotype heterozygosity and geographic distance from Addis Ababa. (TIF) [file pgen.1004530.s001.tif]

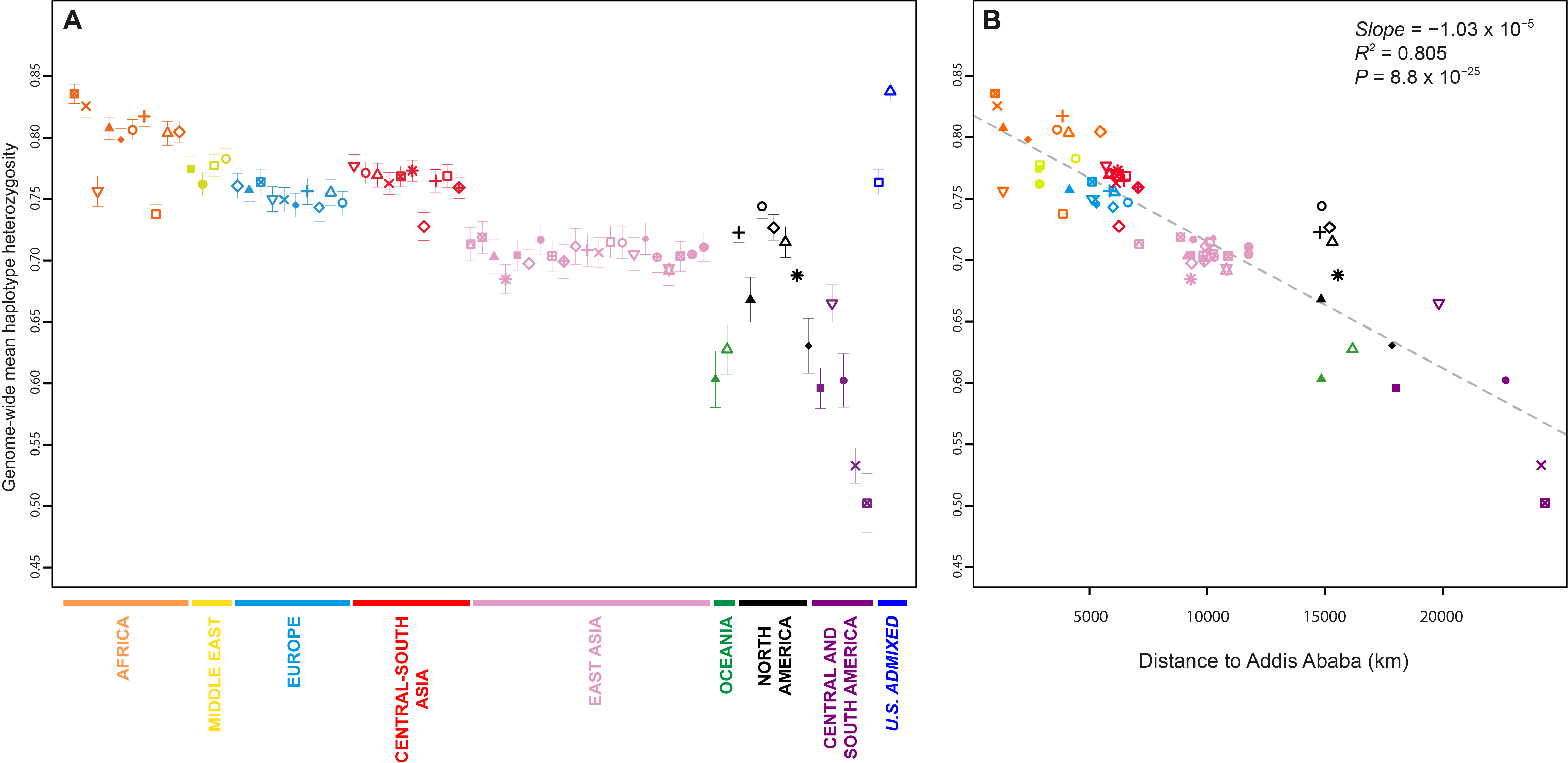

Supplement: Figure S2 — Genome-wide haplotype heterozygosities using haplotype blocks constructed from random SNPs. We define genome-wide haplotypes as random nonoverlapping blocks of 5 to 15 contiguous SNPs in the combined dataset, each block containing the same number of SNPs as the blocks defined previously with all inter-SNP mean recombination rates below 0.5 cM/Mb (with a one-to-one correspondence between blocks). (A) Mean expected haplotype heterozygosity in each population, with standard deviations across the 22 autosomes. (B) The correlation between mean haplotype heterozygosity and geographic distance from Addis Ababa. (TIF) [file pgen.1004530.s002.tif]

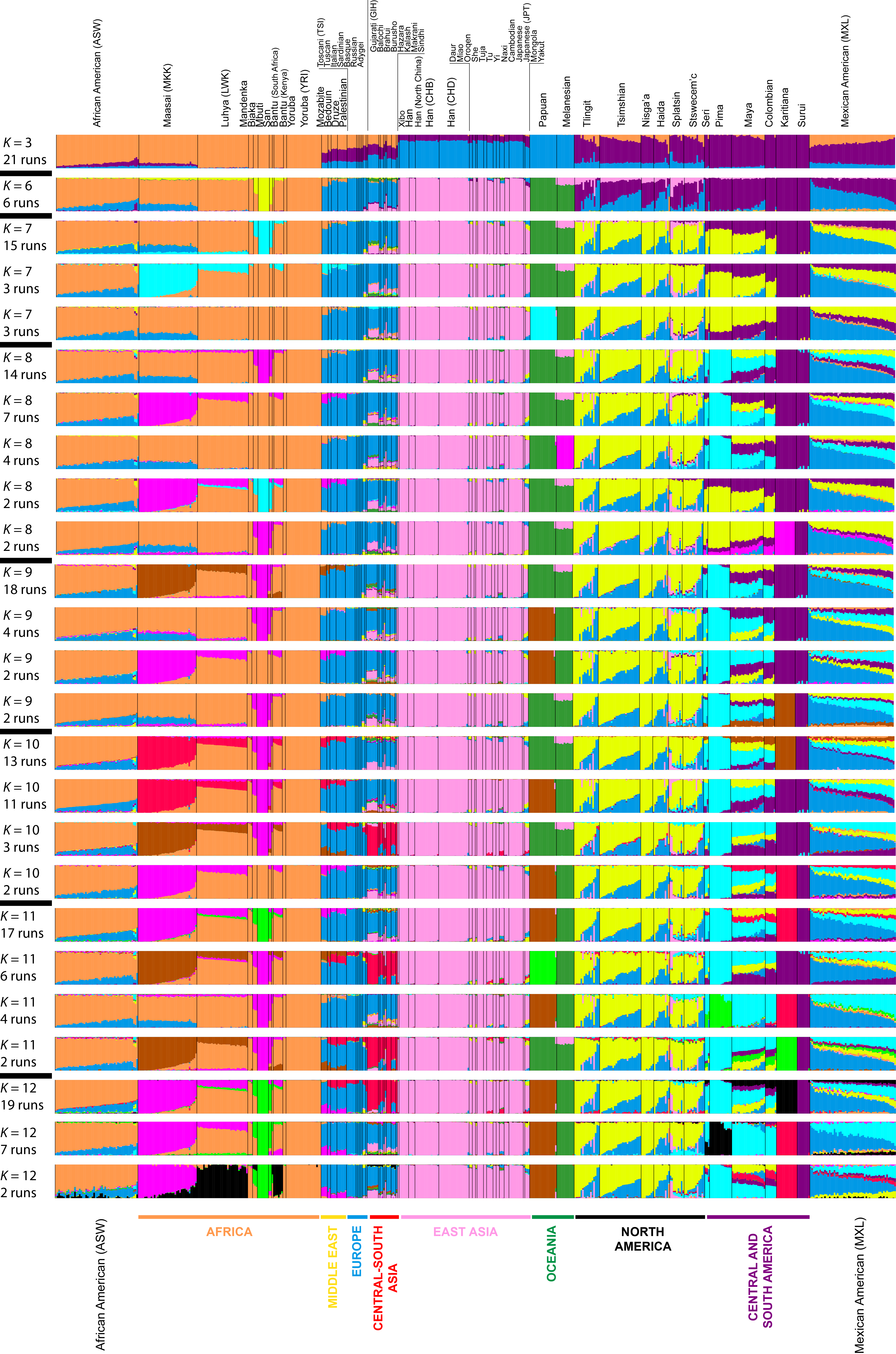

Supplement: Figure S3 — Alternative Admixture structure among worldwide populations for values of K from 2 to 12. Plots are described in Figure 7. (TIF) [file pgen.1004530.s003.tif]

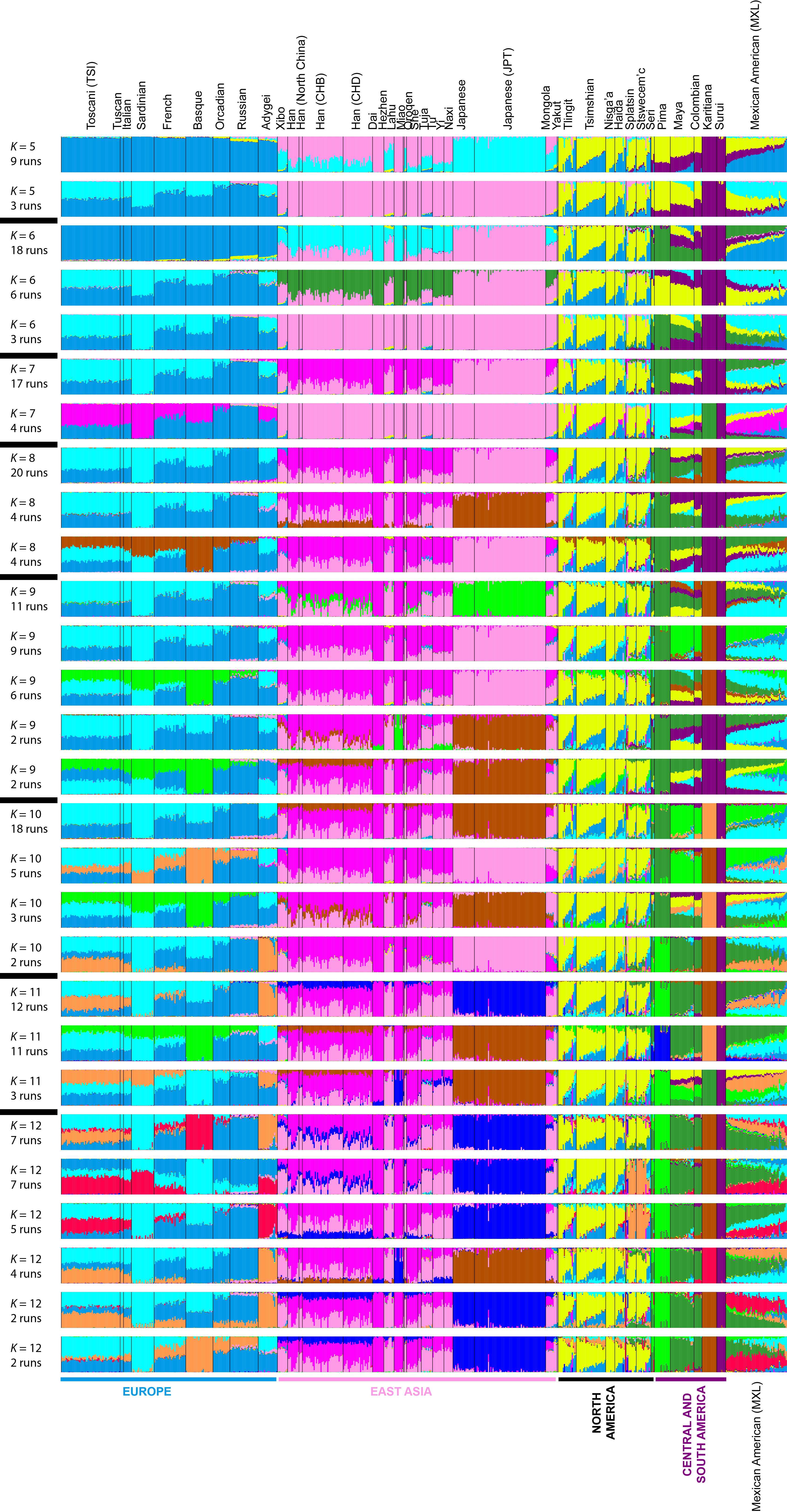

Supplement: Figure S4 — Alternative Admixture structure among European, East Asian, and American populations for values of K from 2 to 12. Plots are described in Figure 7. (TIF) [file pgen.1004530.s004.tif]

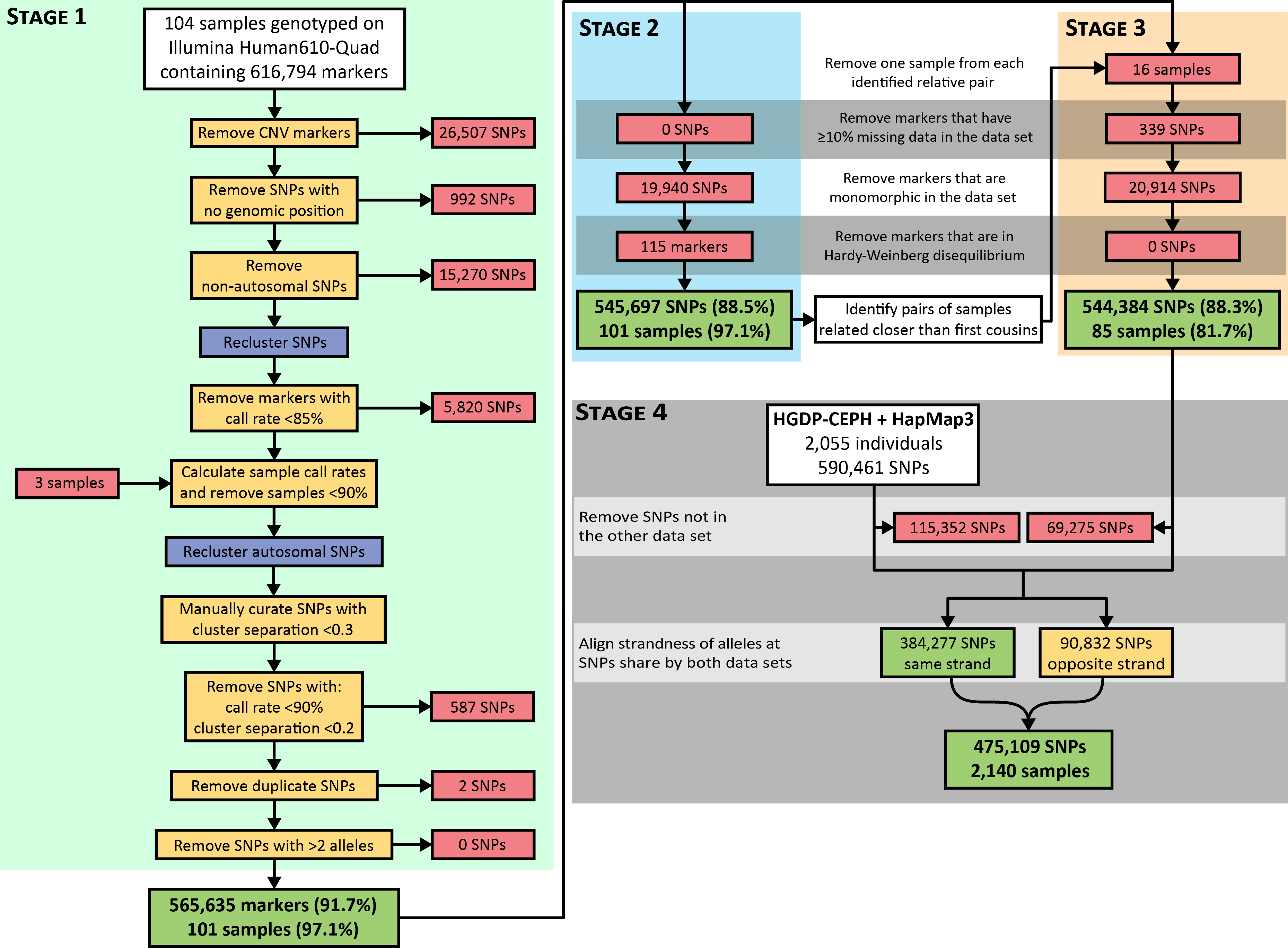

Supplement: Figure S5 — Summary of quality control procedures. (TIF) [file pgen.1004530.s005.tif]

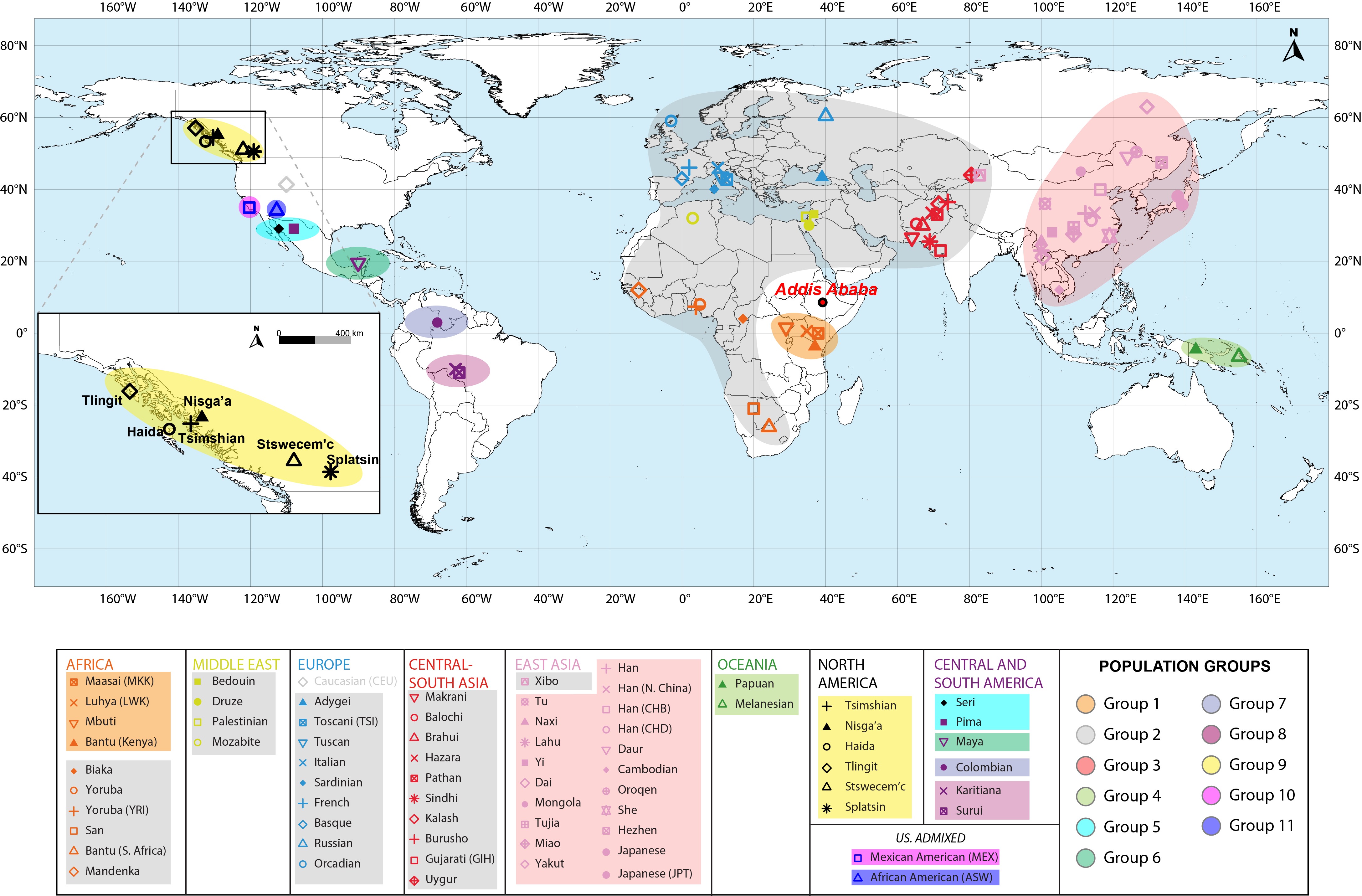

Supplement: Figure S6 — Map of the population groups for analysis worldwide, used in Figure 4B. (TIF) [file pgen.1004530.s006.tif]

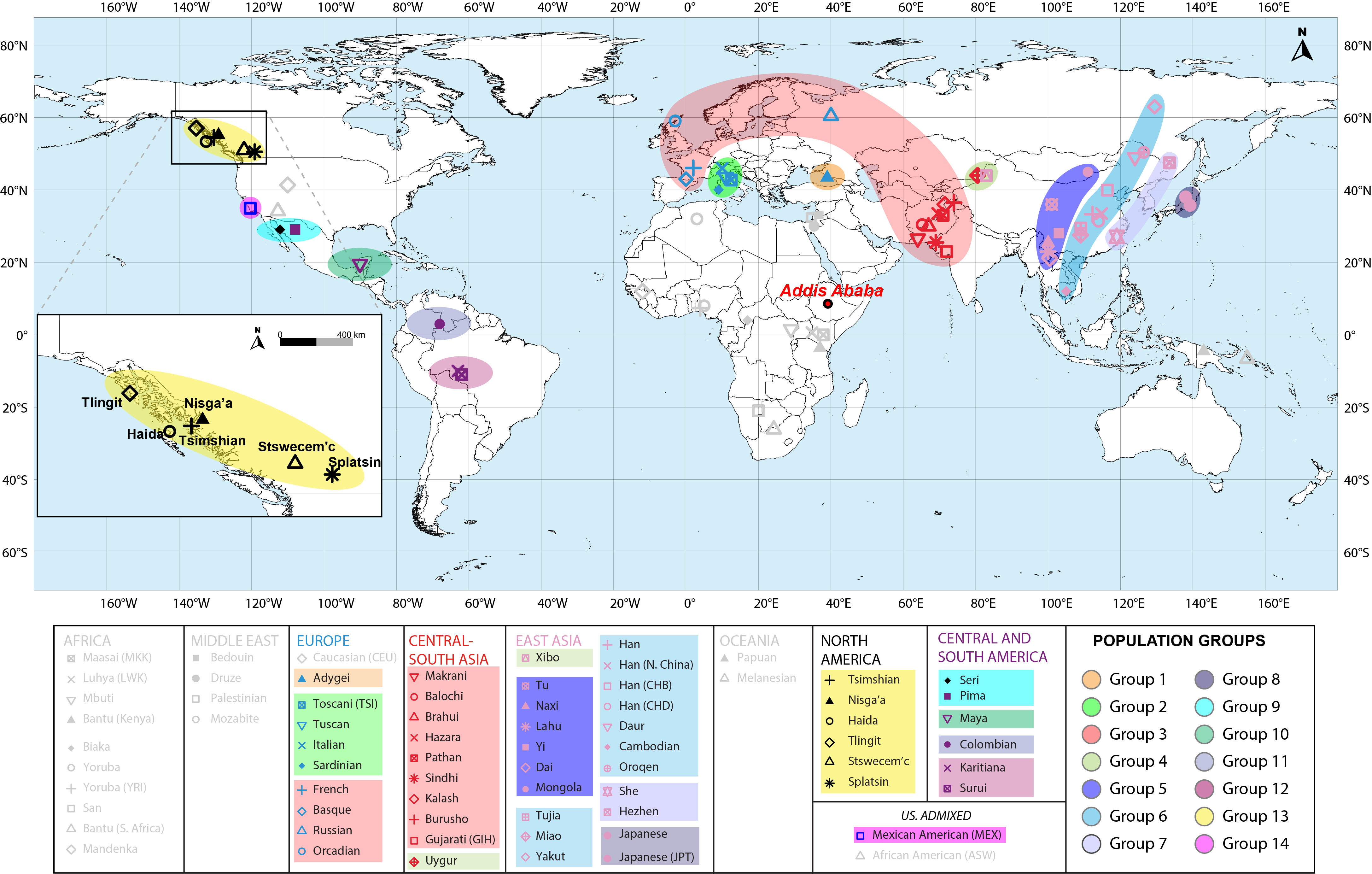

Supplement: Figure S7 — Map of the population groups for analysis with the Eurasian and American populations, used in Figure 5A. (TIF) [file pgen.1004530.s007.tif]

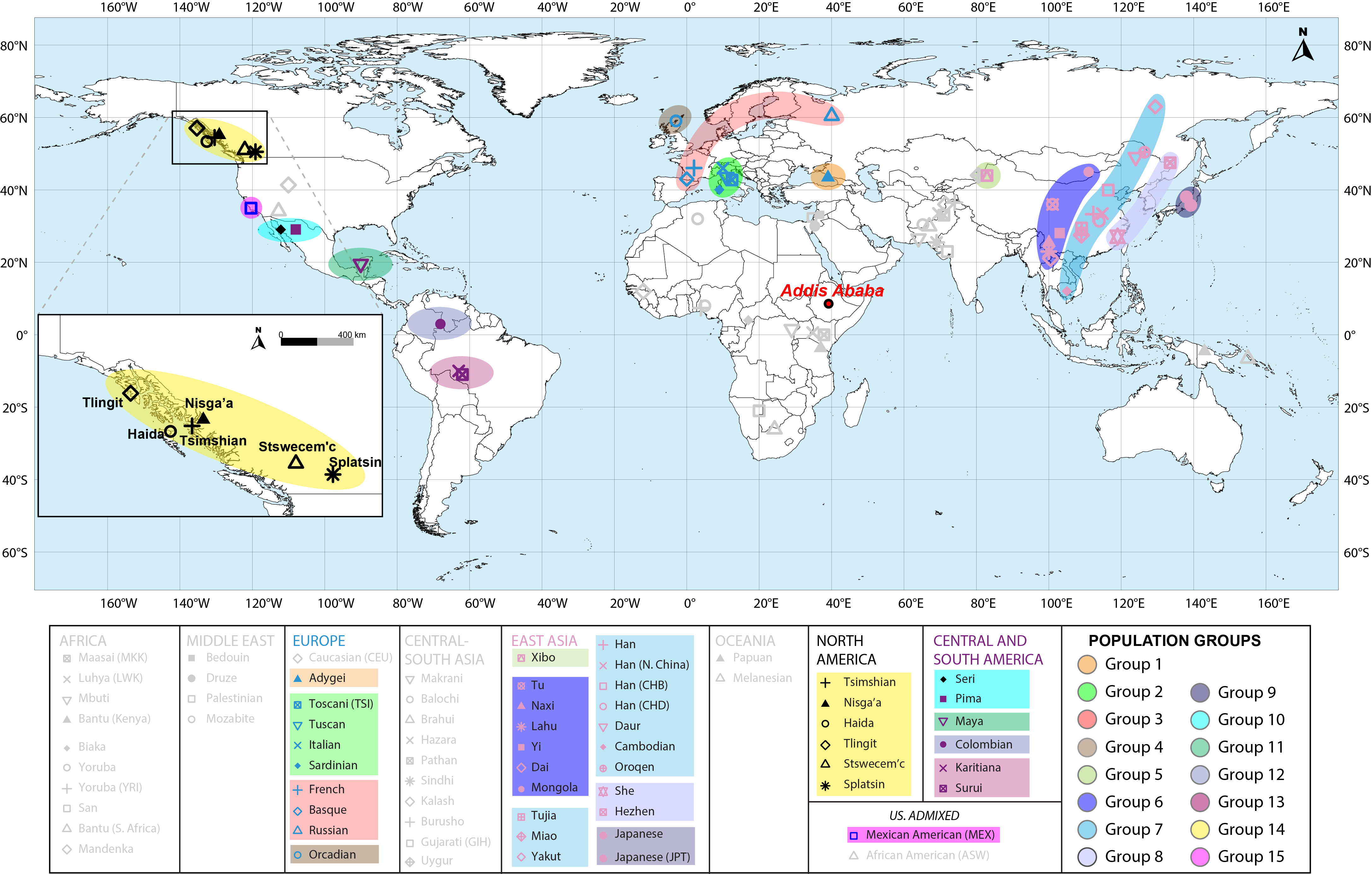

Supplement: Figure S8 — Map of the population groups for analysis with the European, East Asian, and American populations, used in Figure 5B. (TIF) [file pgen.1004530.s008.tif]

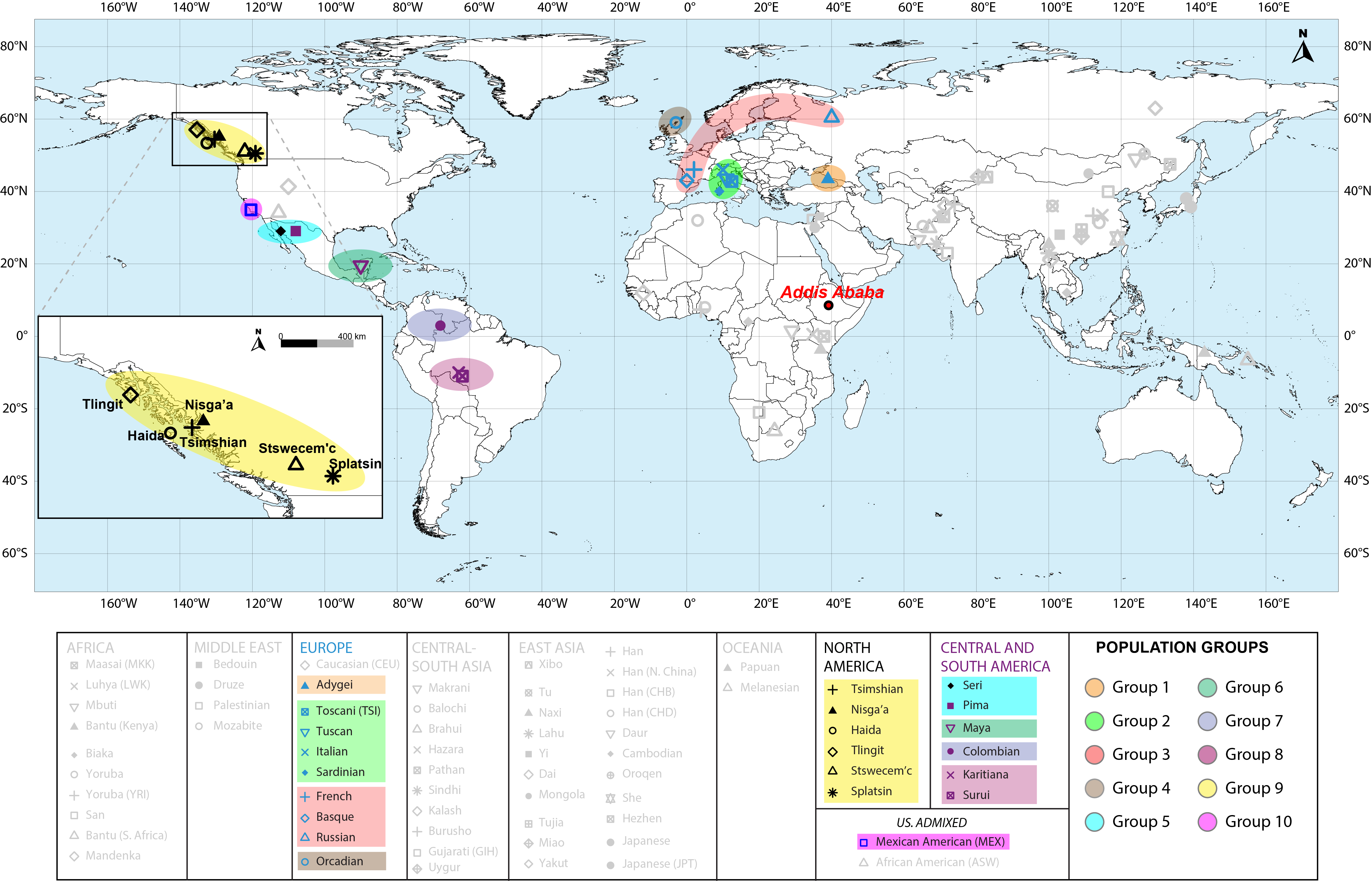

Supplement: Figure S9 — Map of the population groups for analysis with the European and American populations, used in Figure 5C. (TIF) [file pgen.1004530.s009.tif]

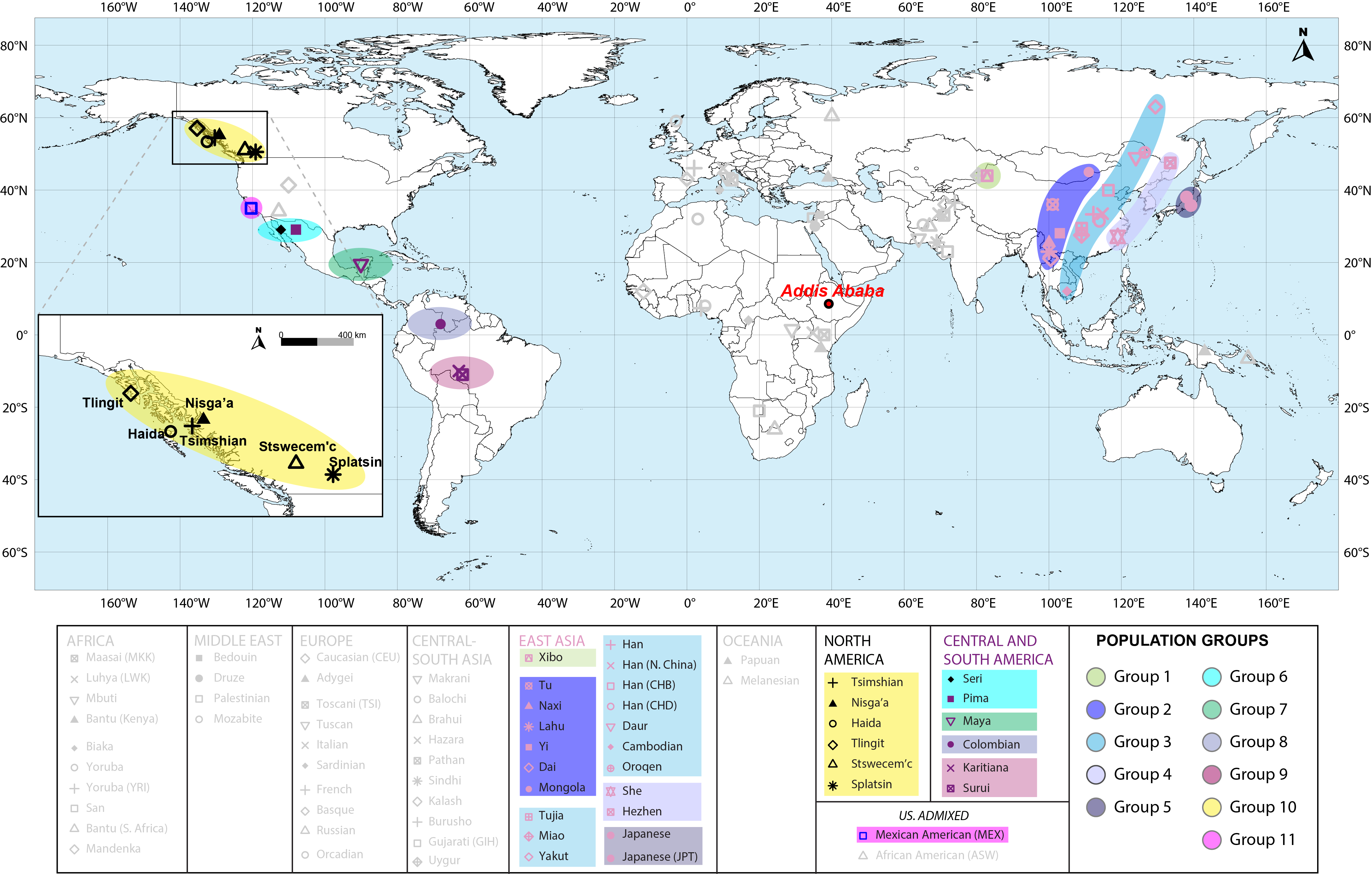

Supplement: Figure S10 — Map of the population groups for analysis with the East Asian and American populations, used in Figure 5D. (TIF) [file pgen.1004530.s010.tif]
