# Supplementary material for: Patterns of Admixture and Population Structure in Native Populations of Northwest North America
Source: PLoS Genet. 2014 Aug 14;10(8):e1004530. doi: 10.1371/journal.pgen.1004530 (PMC4133047; doi:10.1371/journal.pgen.1004530)
Supplement: Table S1 — Populations in the combined dataset. aHapMap Phase III. bHGDP-CEPH. cThis study. dThe distance to Addis Ababa along waypoint routes. eGenome-wide mean haplotype heterozygosity and standard deviation across 22 chromosomes. fThe fraction of missing genotype data among the 475,109 total SNPs in the combined dataset, with the standard deviation taken across individuals within the population. (DOCX) [file pgen.1004530.s011.docx]

| **Continental location** | **Population** | **Number of unrelated individuals** | **Distance to East Africa in kilometers**^d^ | **Haplotype hetero-zygosity (SD)** ^e^ | **Missing data frequency (SD)** ^f^ |
| --- | --- | --- | --- | --- | --- |
| AFRICA | ^a^ Maasai (MKK) | 105 | 1007 | 0.786 (0.008) | 0.0030 (0.0078) |
|  | ^a^ Luhya (LWK) | 99 | 1094 | 0.774 (0.008) | 0.0031 (0.0065) |
|  | ^b^ Mbuti | 13 | 1335 | 0.706 (0.012) | 0.0009 (0.0010) |
|  | ^b^ Bantu (Kenya) | 11 | 1339 | 0.761 (0.010) | 0.0005 (0.0002) |
|  | ^b^ Biaka | 22 | 2385 | 0.751 (0.009) | 0.0008 (0.0008) |
|  | ^b^ Yoruba | 21 | 3630 | 0.756 (0.010) | 0.0004 (0.0003) |
|  | ^a^ Yoruba (YRI) | 140 | 3859 | 0.764 (0.009) | 0.0030 (0.0064) |
|  | ^b^ San | 5 | 3872 | 0.691 (0.014) | 0.0007 (0.0001) |
|  | ^b^ Bantu (S. Africa) | 8 | 4122 | 0.760 (0.009) | 0.0006 (0.0003) |
|  | ^b^ Mandenka | 22 | 5470 | 0.753 (0.009) | 0.0004 (0.0002) |
| MIDDLE EAST | ^b^ Bedouin | 45 | 2845 | 0.716 (0.008) | 0.0003 (0.0003) |
|  | ^b^ Druze | 42 | 2887 | 0.702 (0.009) | 0.0006 (0.0013) |
|  | ^b^ Palestinian | 46 | 2887 | 0.718 (0.007) | 0.0006 (0.0009) |
|  | ^b^ Mozabite | 27 | 4418 | 0.730 (0.009) | 0.0003 (0.0002) |
| EUROPE | ^a^ Caucasian (CEU) | 112 | *-* | 0.700 (0.008) | 0.0023 (0.0049) |
|  | ^b^ Adygei | 17 | 4155 | 0.699 (0.009) | 0.0006 (0.0007) |
|  | ^a^ Toscani (TSI) | 102 | 5118 | 0.703 (0.007) | 0.0022 (0.0045) |
|  | ^b^ Tuscan | 7 | 5118 | 0.698 (0.008) | 0.0003 (0.0002) |
|  | ^b^ Italian | 12 | 5249 | 0.694 (0.007) | 0.0003 (0.0003) |
|  | ^b^ Sardinian | 28 | 5306 | 0.686 (0.007) | 0.0004 (0.0006) |
|  | ^b^ French | 28 | 5857 | 0.696 (0.008) | 0.0007 (0.0013) |
|  | ^b^ Basque | 24 | 6012 | 0.684 (0.009) | 0.0003 (0.0002) |
|  | ^b^ Russian | 25 | 6067 | 0.695 (0.009) | 0.0004 (0.0007) |
|  | ^b^ Orcadian | 15 | 6637 | 0.690 (0.008) | 0.0004 (0.0003) |
| CENTRAL/  SOUTH ASIA | ^b^ Makrani | 25 | 5705 | 0.719 (0.010) | 0.0003 (0.0001) |
|  | ^b^ Balochi | 24 | 5842 | 0.710 (0.008) | 0.0003 (0.0003) |
|  | ^b^ Brahui | 25 | 5842 | 0.709 (0.010) | 0.0004 (0.0003) |
|  | ^b^ Hazara | 22 | 6133 | 0.699 (0.008) | 0.0005 (0.0006) |
|  | ^b^ Pathan | 22 | 6179 | 0.707 (0.009) | 0.0003 (0.0002) |
|  | ^b^ Sindhi | 24 | 6202 | 0.714 (0.009) | 0.0003 (0.0002) |
|  | ^b^ Kalash | 23 | 6254 | 0.669 (0.009) | 0.0003 (0.0002) |
|  | ^b^ Burusho | 25 | 6476 | 0.703 (0.008) | 0.0004 (0.0003) |
|  | ^a^ Gujarati (GIH) | 97 | 6574 | 0.703 (0.008) | 0.0020 (0.0039) |
|  | ^b^ Uygur | 10 | 7072 | 0.699 (0.010) | 0.0002 (0.0001) |
| EAST ASIA | ^b^ Xibo | 9 | 7110 | 0.643 (0.008) | 0.0005 (0.0006) |
|  | ^b^ Tu | 10 | 8868 | 0.648 (0.010) | 0.0002 (0.0002) |
|  | ^b^ Naxi | 8 | 9131 | 0.634 (0.011) | 0.0004 (0.0005) |
|  | ^b^ Lahu | 8 | 9300 | 0.615 (0.011) | 0.0002 (0.0001) |
|  | ^b^ Yi | 10 | 9329 | 0.636 (0.011) | 0.0002 (0.0001) |
|  | ^b^ Dai | 10 | 9344 | 0.627 (0.009) | 0.0010 (0.0013) |
|  | ^b^ Mongola | 10 | 9409 | 0.647 (0.010) | 0.0002 (0.0001) |
|  | ^b^ Tujia | 10 | 9833 | 0.631 (0.009) | 0.0002 (0.0001) |
|  | ^b^ Miao | 10 | 9875 | 0.627 (0.010) | 0.0005 (0.0007) |
|  | ^b^ Yakut | 25 | 9920 | 0.642 (0.009) | 0.0006 (0.0014) |
|  | ^b^ Han | 34 | 10133 | 0.633 (0.010) | 0.0007 (0.0025) |
|  | ^b^ Han (N. China) | 10 | 10133 | 0.636 (0.008) | 0.0002 (0.0001) |
|  | ^a^ Han (CHB) | 137 | 10133 | 0.638 (0.008) | 0.0040 (0.0095) |
|  | ^a^ Han (CHD) | 106 | 10133 | 0.637 (0.008) | 0.0041 (0.0091) |
|  | ^b^ Daur | 9 | 10213 | 0.638 (0.011) | 0.0003 (0.0003) |
|  | ^b^ Cambodian | 10 | 10261 | 0.650 (0.008) | 0.0012 (0.0026) |
|  | ^b^ Oroqen | 9 | 10294 | 0.633 (0.010) | 0.0002 (0.0001) |
|  | ^b^ She | 10 | 10818 | 0.621 (0.009) | 0.0007 (0.0011) |
|  | ^b^ Hezhen | 9 | 10896 | 0.631 (0.011) | 0.0003 (0.0003) |
|  | ^b^ Japanese | 28 | 11762 | 0.628 (0.013) | 0.0006 (0.0013) |
|  | ^a^ Japanese (JPT) | 113 | 11762 | 0.633 (0.010) | 0.0031 (0.0055) |
| OCEANIA | ^b^ Papuan | 17 | 14843 | 0.537 (0.021) | 0.0009 (0.0016) |
|  | ^b^ Melanesian | 11 | 16168 | 0.563 (0.017) | 0.0007 (0.0012) |
| NORTH AMERICA | ^c^ Tsimshian | 26 | 14773 | 0.658 (0.012) | 0.0003 (0.0007) |
|  | ^c^ Nisga’a | 8 | 14833 | 0.609 (0.017) | 0.0118 (0.0235) |
|  | ^c^ Haida | 10 | 14857 | 0.687 (0.008) | 0.0049 (0.0076) |
|  | ^c^ Tlingit | 16 | 15194 | 0.665 (0.010) | 0.0068 (0.0132) |
|  | ^c^ Stswecem’c | 13 | 15311 | 0.654 (0.012) | 0.0106 (0.0264) |
|  | ^c^ Splatsin | 9 | 15548 | 0.624 (0.012) | 0.0131 (0.0297)) |
| CENTRAL & SOUTH AMERICA | ^c^ Seri | 3 | 17846 | 0.572 (0.029) | 0.0537 (0.0238) |
|  | ^b^ Pima | 14 | 18016 | 0.526 (0.021) | 0.0004 (0.0004) |
|  | ^b^ Maya | 21 | 19826 | 0.596 (0.019) | 0.0008 (0.0017) |
|  | ^b^ Colombian | 7 | 22662 | 0.536 (0.025) | 0.0004 (0.0004) |
|  | ^b^ Karitiana | 13 | 24177 | 0.473 (0.019) | 0.0003 (0.0002) |
|  | ^b^ Surui | 8 | 24330 | 0.445 (0.021) | 0.0002 (0.0001) |
| US ADMIXED | ^a^ African American (ASW) | 52 | *-* | 0.790 (0.006) | 0.0012 (0.0011) |
|  | ^a^ Mexican American (MXL) | 54 | *-* | 0.702 (0.012) | 0.0034 (0.0051) |
